# The association between posttraumatic stress disorder and migraine: A systematic review

**DOI:** 10.1111/head.70058

**Published:** 2026-02-18

**Authors:** Lucie Nitsche, Jasmin Helbach, Sarah Stubenrauch, Meret Lakeberg, Carsten Bantel, Falk Hoffmann

**Affiliations:** ^1^ Department of Health Services Research, School of Medicine and Health Sciences Carl von Ossietzky Universität Oldenburg Oldenburg Germany; ^2^ University Department of Anesthesiology, Critical Care, Emergency and Pain Medicine Klinikum Oldenburg Oldenburg Germany

**Keywords:** etiology, evidence synthesis, headache, mental disorders, neurological disorders, pain

## Abstract

**Objectives:**

The aim of this systematic review was to assess the magnitude of the association between posttraumatic stress disorder (PTSD) and migraine by synthesizing prevalence and incidence data from relevant studies.

**Background:**

Migraine is associated with reduced quality of life and an increased risk for psychiatric comorbidities. Recent evidence indicates a growing relevance of PTSD in this context.

**Methods:**

A comprehensive literature search was conducted in MEDLINE (via PubMed), EMBASE (via Elsevier), and PsycInfo (via EBSCOhost) from inception to November 22, 2024. Studies were eligible if they included adult populations and reported migraine prevalence or incidence in PTSD and non‐PTSD groups. No restrictions were made regarding setting, language, or publication date. Two independent reviewers conducted study selection and data extraction. Results were narratively synthesized, and study quality was assessed using the Joanna Briggs Institute checklists.

**Results:**

Of 12,801 records, 11 studies from five countries were included. Study populations included military personnel, general population, nurses, pregnant women, and students. Migraine prevalence (*n* = 9) was higher in individuals with PTSD (6.5%–46.5%) compared to those without (1.4%–25.8%), as were incidence estimates (*n* = 1; 48.5% vs. 39.5%) and incidence rates (*n* = 1; 5.74 vs. 1.22). All studies reported a positive association between PTSD and migraine, with ratios ranging from 1.2 to 4.7.

**Conclusion:**

This systematic review demonstrates a strong association between PTSD and migraine that extends beyond predisposed populations. These findings highlight the need for systematic trauma assessment in patients with chronic headache disorders. Future research should further explore this bidirectional interaction in population‐based samples.

PROSPERO study registration: CRD4202461764.

## Plain Language Summary

Previous research suggests that migraine is often linked to posttraumatic stress disorder (PTSD). We reviewed and summarized literature that reports how common migraine is and how many new cases occur among people with and without PTSD, and our results showed that people with PTSD are more likely to experience migraine than those without PTSD, regardless of population or study type. Healthcare providers should consider assessing for history of traumatic stress in patients with migraine because this may help to reduce long‐term disability.

**Abbreviations**
CI: confidence interval
CS: central sensitization
DSM: Diagnostic and Statistical Manual of Mental Disorders
HR: hazard ratio
ICD: International Classification of Diseases
ICHD: International Classification of Headache Disorders
JBI: Joanna Briggs Institute
OR: odds ratio
PRISMA: Preferred Reporting Items for Systematic Reviews and Meta‐analyses
PROSPERO: Prospective Register of Systematic Reviews study registration
PTSD: posttraumatic stress disorder
PY: person years
RR: relative risk

## INTRODUCTION

Migraine, which affects 14%–15% of adults worldwide, is one of the most common neurological disorders.^1^ It is characterized by recurrent, severe headache, often accompanied by photophobia and phonophobia, and is associated with motion sensitivity symptoms, including nausea, vomiting, and vestibular dysfunction.^2^, ^3^ Migraine significantly impairs quality of life, reduces productivity, and is among the leading causes of disability, placing a substantial burden on the economy and society.^4^, ^5^, ^6^ Beyond its physical impact, migraine is strongly associated with psychiatric conditions,^7^, ^8^ indicating a complex interplay between neurological and psychological factors.^9^

Prior research on psychiatric comorbidities in migraine primarily focused on mood disorders such as anxiety or depression.^10^, ^11^, ^12^, ^13^ More recently, interest has grown in examining trauma and posttraumatic stress disorder (PTSD) in the context of migraine.^14^, ^15^, ^16^, ^17^ PTSD is triggered by severe traumatic events and is characterized by intrusive memories and heightened psychological distress.^18^ It frequently co‐occurs with chronic pain syndromes, like chronic low back pain or fibromyalgia.^19^, ^20^ Epidemiological studies estimate that 20%–30% of individuals with migraine also meet diagnostic criteria for PTSD,^21^, ^22^ which indicates a substantial overlap between the two conditions.

Nevertheless, the extent to which the two disorders influence each other remains insufficiently explored. Migraine prevalence in individuals with PTSD is often reported as a secondary outcome rather than the primary focus, with prevalence estimates varying widely due to differences in study populations because many focused on specific traumatized groups (e.g., veterans) and variations in methodologies and diagnostic criteria.^23^, ^24^, ^25^ The fact that only few studies directly compare migraine prevalence between populations with PTSD and those without PTSD further complicates the interpretation of the magnitude of this association. Overall, findings remain heterogeneous, and a comprehensive synthesis of the literature is currently lacking.

This review aims to address this gap by systematically analyzing the existing literature and assessing migraine prevalence and incidence in individuals with PTSD versus without PTSD. As a secondary objective, we examined multiple adjusted association measures reported in the literature to further evaluate the magnitude of the PTSD–migraine relationship.

## METHODS

This systematic review was conducted following the methodological framework described by the Joanna Briggs Institute (JBI) for systematic reviews of etiology and risk^26^ and was reported in accordance with the Preferred Reporting Items for Systematic Reviews and Meta‐Analyses (PRISMA) statement.^27^ A protocol has been registered in the International Prospective Register of Systematic Reviews (PROSPERO) (CRD42024617648).

### Data sources and search

A comprehensive literature search was conducted in MEDLINE (via PubMed), EMBASE (via Elsevier), and PsycInfo (via EBSCOhost) from inception to November 22, 2024. The systematic search strategy is presented in the Supporting Information (Table S1). A forward and backward citation analysis was conducted on the reports of the included studies on February 27, 2025, using the Web of Science Core Collection. This analysis was repeated iteratively on newly identified eligible references until no further studies could be identified.

### Eligibility criteria

The eligibility criteria were defined by the population, exposure, outcome format for reviews assessing data on etiology and risk.^26^ Studies were included regardless of their context or setting, and no language or date restrictions were applied.

### Population

We included studies that focused on adult populations aged 18 years and older, irrespective of underlying etiological factors or comorbid conditions. Studies exclusively investigating pediatric or adolescent populations (<18 years) were excluded. Eligible studies required a minimum sample size of 100 participants, with at least 50 individuals diagnosed with PTSD and a further 50 without PTSD. This restriction was intended to exclude pilot studies and studies with a very small sample size because these are often subject to a certain degree of uncertainty, frequently refer to selective samples, and do not provide robust estimates.

### Exposure

We included studies that based the PTSD diagnosis on established clinical diagnostic criteria (e.g., Diagnostic and Statistical Manual of Mental Disorders [DSM] or International Classification of Diseases and related Health Problems [ICD]), self‐reports (e.g., PTSD scale), or claims data. Given our interest in examining both the absolute and relative association between PTSD and migraine, only studies enabling a comparison of individuals diagnosed with PTSD to those without PTSD (e.g., healthy controls, individuals without PTSD, or with other specific health conditions) were included.

### Outcome

The primary outcome of interest was the diagnosis of migraine, based on clinical diagnostic criteria (e.g., ICHD or ICD), self‐reported measures (e.g., ID‐Migraine screener), or claims data. Studies examining other headache disorders (e.g., tension‐type headache, cluster headache, hypnic headache) or those that reported migraine as part of some other diagnosis without presenting individual estimators were excluded. Only studies reporting migraine prevalence or incidence were eligible.

As a secondary outcome, adjusted association measures between PTSD and migraine were assessed from the selected studies. These included odds ratios (OR), hazard ratios (HR), or relative risk (RR), accounting for potential confounders.

### Study design

Regarding the study type, observational study designs, including prospective and retrospective cohort studies, case–control studies, and cross‐sectional studies, were eligible if they reported prevalence or incidence estimates of migraine. Randomized controlled trials were excluded from the analysis. However, exceptions were made for randomized controlled trials that included cross‐sectional analyses of baseline data, specifically examining the prevalence of migraine in individuals with PTSD compared to those without. Additionally, review articles, editorials, PhD theses, and conference abstracts were excluded.

### Study selection and data

Search results were imported into EndNote (version 20, Clarivate, Philadelphia, PA, USA). After duplicate removal, the results were transferred to Rayyan QCRI for systematic screening. All titles and abstracts were screened independently by two reviewers. LN screened all records, whereas SS, ML, and FH shared the role of the second reviewer. This was followed by a full‐text screening for studies that met the inclusion criteria, conducted independently by two reviewers, maintaining the same allocation of tasks as in the previous step. To ensure inter‐reviewer consistency, a calibration with 100 titles and abstracts was carried out first. Relevant data was extracted by one reviewer (LN) and cross‐checked and recalculated by a second reviewer (SS). Authors of the included studies were only contacted for clarification purposes, such as verification of numbers or methodological details.

Any inconsistencies or discrepancies during the screening or the extraction process were either resolved through discussion or by a third reviewer (FH, JH). Data were directly transferred into the results tables shown below. Key extracted data included study characteristics (author, year of publication, study location, design, and setting), sample size, demographic information (age and sex), PTSD and migraine diagnostic criteria, prevalence or incidence data, and adjusted association measures, along with the relevant confounding variables.

### Quality assessment

The methodological quality of the studies included in the review was independently assessed by two reviewers (LN, ML) using the JBI critical appraisal checklists for cross‐sectional and cohort studies, which were adapted to align with the research question of this review (Supporting Information Tables S2 and S3). Prior to conducting the assessments, the quality evaluation process was piloted on two studies to calibrate the reviewers. Any disagreements between the reviewers were resolved through discussion or by consulting a third reviewer (FH, JH).

### Data synthesis

Data were synthesized descriptively. Given the anticipated heterogeneity in study designs, populations, and measurement methodologies for both the exposure and outcome variables, a meta‐analysis was not conducted. If the prevalence or incidence of PTSD or migraine was not explicitly reported in the text or tables, it was calculated using data extracted from the respective studies (Supporting Information S4). All calculations were conducted in Microsoft Excel (Version 16.99, Microsoft Corp., Redmond, WA).

If multiple articles based on the same data were included, these were linked and reported at the study level.

## RESULTS

### Study selection

**FIGURE 1:**
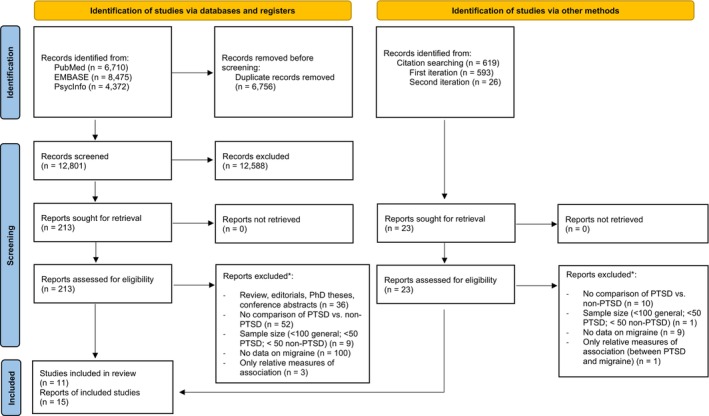
PRISMA flow diagram illustrating study selection. Adapted from: Page et al.^27^ *The assignment of the exclusion was done hierarchically in the order presented. PRISMA, Preferred Reporting Items for Systematic Reviews and Meta‐analyses. [Color figure can be viewed at wileyonlinelibrary.com]

The database search yielded 19,557 records (Figure 1). Following deduplication, a total of 12,801 titles and abstracts were screened, of which 213 full texts were read. Thirteen articles met the inclusion criteria.^28^, ^29^, ^30^, ^31^, ^32^, ^33^, ^34^, ^35^, ^36^, ^37^, ^38^, ^39^, ^40^ For these articles, a forward and backward citation analysis was performed, yielding 593 records, with one article meeting the inclusion criteria.^41^ Based on this article, a second round of citation analysis was conducted, which identified 25 records via Web of Science, none of which were eligible. However, an article from the reference list,^42^ not indexed in Web of Science, did meet the inclusion criteria and was additionally included.

Overall, 11 studies reported in 15 articles were included in this review. All included articles were published in English. Articles excluded during the full‐text screening, together with reasons for exclusion, are listed in the Supporting Information (Table S5).

### Study and participant characteristics

The included studies were conducted in the United States (*n* = 7), Peru (*n* = 1), Taiwan (*n* = 1), Canada (*n* = 1), and Israel (*n* = 1) (Table 1). Eight were cross‐sectional and three were cohort studies, although a cohort study reported cross‐sectional data. Data collection spanned from 1989 to 2020, with one study not reporting the data collection period.

**TABLE 1:** Baseline characteristics of the included studies.

Author(s) + (Year)	Country	Data source	Year of data	Study design	Sample size	Population	Mean age, proportion female
*Prevalence studies*							
Barer et al.^28^	Israel	Claims data (MHS)	2000–2015	Cohort study	*n* = 16,672	Members of Maccabi Healthcare Services	55.8 years, female: 48.4%
El‐Gabalawy et al.^39^ with companion reports Nichter et al.^36^ Stefanovics et al.^37^	USA	National Health and Resilience in Veterans Study (NHRVS)	2011	Cross‐sectional	*n* = 2933^a^	U.S. adult veterans	N/R, female: 11.8%
Friedman et al.^29^	Peru	Pregnancy Outcomes, Maternal and Infant Study (PrOMIS)	2012–2014	Cross‐sectional	*n* = 2922	Pregnant women	28.1 years, female: 100%
Friedman et al.^30^	USA	Nationwide Inpatient Sample (NIS) database	2007–2012	Cross‐sectional	*n* = 156,172,826	Adult participants with hospital discharges	57.2 years, female: 59.9%
Gasperi et al.^31^	USA	Vietnam Era Twin (VET) Registry	2010–2012	Cross‐sectional	*n* = 4554^b^	Male–male twin pairs born between 1939 and 1957, Vietnam veterans	61.1 years, female: 0%
Herbert et al.^32^	USA	Million Veteran Program (MVP)	2011–2020	Cross‐sectional	*n* = 338,217	U.S. adult veterans	67.2 years, female: 7.9%
Rao et al.^33^ with companion report: Peterlin et al.^38^	USA	National Comorbidity Survey Replication (NCS‐R)	2001–2003	Cross‐sectional	*n* = 5083	U.S. general adult population	44.8 years, female: 51.9%^d^
Smitherman and Kolvivas^34^	USA	Web‐administered battery	N/R	Cross‐sectional	*n* = 1051	Undergraduate students (18–30 yrs.)	18.9 years, female: 63.1%
Vun et al.^35^	Canada	Canadian Forces Mental Health Survey (CFMHS)	2013	Cross‐sectional	*n* = 6696	Canadian regular force members	35.4 years, female: 13.9%
*Incidence studies*							
Crowe et al.^41^	USA	Nurse's Health Study II (NHS II)	1989–2020	Cohort study	*n* = 28,815^c^	Female nurses	64.0 years, female: 100%
Huang et al.^42^ with companion report Chan et al.^40^	Taiwan	Claims data (NHIRD)	2002–2011	Cohort study	*n* = 28,220	Members of Taiwan's National Health Insurance	34.6 years, female: 75.6%

Abbreviations: MHS, Maccabi Healthcare Services; NHIRD, National Health Insurance Research Database; N/R, not reported.

^a^ Sample size differs from the sample size reported in the original study (*n* = 3157); for more details see Supporting Information S4.

^b^ Sample size differs from the sample size reported in the original study (*n* = 4680); for more details see Supporting Information S4.

^c^ Sample size differs from the sample size reported in the original study (*n* = 33,327); for more details see Supporting Information S4.

^d^ Independent calculation based on figures in the respective study (Supporting Information S4).

Study populations varied considerably. Four studies examined military populations, two analyzed data from a national health insurance registry, and two presented findings from the U.S. general population. The remaining studies focused on female nurses (*n* = 1), pregnant women (*n* = 1), and undergraduate students (*n* = 1). Sample sizes ranged from 1051 to 156,172,826, with female proportions varying between 0% and 100% (*n* = 11) and mean ages ranging from 18.9 to 67.2 years (*n* = 10).

### Quality assessment

Among the nine cross‐sectional studies, eight defined eligibility criteria for participant selection (Supporting Information Tables S2 and S3). Detailed participant and setting descriptions were provided in two out of the nine studies. Regarding PTSD assessment, two‐thirds of the studies used validated and reliable tools. In five out of nine studies, migraine assessment was based on established clinical diagnostic criteria, self‐reports, or claims data, and was therefore classified as valid according to the JBI critical appraisal checklists. The remaining studies did not provide detailed information on migraine assessment or applied non‐standardized diagnostic instruments.

In the two cohort studies, both recruited control groups similar to their study populations, used validated PTSD measures, and had a follow‐up period of at least 24 months. Migraine was assessed using validated methods in one of these studies. Neither study reported specific numbers or details regarding loss to follow‐up management.

### PTSD prevalence

In most studies, PTSD was diagnosed using different versions of the PTSD checklist (*n* = 5), a self‐report questionnaire based on DSM criteria (Table 2). Two studies used the World Health Organization Composite International Diagnostic Interview (*n* = 2), an assessment instrument also based on DSM criteria. Other studies assessed PTSD according to ICD classifications (*n* = 3). One study based the PTSD diagnosis on participants' self‐reported history of PTSD diagnosis (*n* = 1). Overall PTSD prevalence in the included studies ranged from 0.5%–50.0%, with the highest estimates reported in Israeli health insurance data and the lowest among hospital discharges. In studies from military context (*n* = 4), PTSD prevalence ranged from 5.3% to 19.9%.

**TABLE 2:** Main results of included studies on migraine and PTSD: Diagnostic criteria, prevalence and incidence estimates.

Author(s) + (Year)	Country	Population and sample size	PTSD diagnostic criteria	Overall PTSD prevalence	Migraine diagnostic criteria	Overall migraine prevalence/incidence	Migraine prevalence/incidence in PTSD	Migraine prevalence/incidence in non‐PTSD‐group	Ratio migraine in PTSD vs. non‐PTSD
*Prevalence studies*									
Barer et al.^28^	Israel	Members of Maccabi Healthcare Services, *n* = 16,672	ICD‐9	50.0% (*n* = 8336)	N/R	5.6%^d^ (*n* = 938)	6.8% (*n* = 570)	4.4% (*n* = 368)	1.5
El‐Gabalawy et al.^39^	USA	U.S. adult veterans, *n* = 2933^a^	PCL‐S, DSM‐IV	16.1%^d^ (*n* = 471)	Self‐reported history of diagnosis	6.3%^d^ (*n* = 186)	14.0%^d^ (*n* = 66)	4.9%^d^ (*n* = 120)	2.9
Friedman et al.^29^	Peru	Pregnant women, *n* = 2922	PCL‐C, DSM‐IV	37.4% (*n* = 1093)	ICHD‐3 beta criteria	33.5%^d^ (*n* = 979)	46.5%^d^ (*n* = 508)	25.8%^d^ (*n* = 471)	1.8
Friedman et al.^30^	USA	Hospital discharges, *n* = 156,172,826	ICD‐9	0.5% (*n* = 840,338)	ICD‐9	1.4%^d^ (*n* = 2,190,305)	6.5%^d^ (*n* = 54,429)	1.4%^d^ (*n* = 2,135,876)	4.6
Gasperi et al.^31^	USA	Male twin veterans, *n* = 4554^b^	PCL‐C, DSM‐IV	19.9%^d^ (*n* = 908)	Self‐reported history of diagnosis	7.6% (*n* = 347)^d^	17.3% (*n* = 157^d^)	5.2% (*n* = 190^d^)	3.3
Herbert et al.^32^	USA	U.S. adult veterans, *n* = 338,217	Self‐reported history of diagnosis	16.7% (*n* = 56,461)	Self‐reported history of diagnosis	8.9% (*n* = 30,080)	19.4%^d^ (*n* = 10,970)	6.8%^d^ (*n* = 19,110)	2.9
Rao et al.^33^	USA	U.S. general adult population, *n* = 5083	WHO‐CIDI, DSM‐IV	6.1%^d^ (*n* = 312)	Self‐Report, ICHD‐2	6.0%^d^ (*n* = 304)	21.8%^d^ (*n* = 68)	4.9%^d^ (*n* = 236)	4.4
Smitherman and Kolvivas^34^	USA	Undergraduate students, *n* = 1051	PCL‐C, DSM‐IV	17.5%^d^ (*n* = 184)	Self‐Report, SDIH‐R, ICHD‐2	28.5% (*n* = 300)	41.8%^d^ (*n* = 77)	25.7%^d^ (*n* = 223)	1.6
Vun et al.^35^	Canada	Canadian Regular Force members, *n* = 6696	WHO‐CIDI, DSM‐IV	5.3% (*n* = 348)	Self‐reported history of diagnosis	9.1% (*n* = 613)	24.7%^d^ (*n* = 86)	8.3%^d^ (*n* = 527)	3.0
*Incidence studies*									
Crowe et al.^41^	USA	Female nurses, *n* = 28,815^c^	PCL‐5, DSM‐V	7.9%^d^ (*n* = 2279)	Self‐reported history of diagnosis	40.2%^d^ (*n* = 11,593)	48.5% (*n* = 1105)	39.5%^d^ (*n* = 10,488)	1.2
Huang et al.^42^	Taiwan	Members of Taiwan's National Health Insurance, *n* = 28,220	ICD‐9	20.0%^d^ (*n* = 5644)	ICD‐9	2.10 per 1000 PY^d^	5.74 per 1000 PY	1.22 per 1000 PY	4.7

Abbreviations: CIDI, Composite International Diagnostic Interview; DSM‐IV, Diagnostic and Statistical Manual of Mental Disorders (2013); ICD‐9, International Classification of Diseases and related Health Problems 9th Revision; ICHD, International Classification of Headache; PCL‐C, PTSD checklist‐Civilian Version; PCL‐S, PTSD checklist‐Specific Stressor Version; PTSD, posttraumatic stress disorder; PY, person‐years; SDIH‐R, Structured Diagnostic Interview for Headache‐Revised; WHO, World Health Organization.

^a^ Sample size differs from the sample size reported in the original study (*n* = 3157); for more details see Supporting Information S4.

^b^ Sample size differs from the sample size reported in the original study (*n* = 4680); for more details see Supporting Information S4.

^c^ Sample size differs from the sample size reported in the original study (*n* = 33,327); for more details see Supporting Information S4.

^d^ Independent calculation based on figures reported in the respective study (for more details see Supporting Information S4).

### Migraine prevalence and incidence

Migraine assessment methods varied across studies (Table 2). In most studies, migraine was assessed relying on participants' self‐reported history of migraine diagnosis (*n* = 5). Other studies assessed migraine using diagnostic criteria from the ICHD (*n* = 3), either alone or in combination with other methods such as the Structured Diagnostic Interview for Headache (*n* = 1) or self‐reports of ever having migraine (*n* = 2). Two studies based the diagnosis of migraine on ICD codes, and one did not specify the diagnostic approach. Overall reported migraine prevalence ranged from 1.4% in hospital discharge data to 33.5% among pregnant women. On average, studies based on civilian population samples reported higher prevalences than those conducted in military populations. One study provided estimates of migraine incidence (40.2%), and one provided incidence rates per 1000 person‐years (PY) (2.10 per 1000 PY).

### Migraine prevalence and incidence in individuals with versus without PTSD

Direct comparisons of migraine prevalence and incidence estimates between PTSD and non‐PTSD groups showed ratios between 1.2 and 4.7, with a median of 2.9 (Table 2). The highest ratio was observed among members of Taiwan's National Health Insurance, whereas the lowest was found among female nurses. On average, military‐related studies produced marginally higher ratios (2.9–3.3) compared to those conducted in non‐military populations (1.2–4.7). Across all studies, migraine prevalence was consistently higher among individuals with PTSD, ranging from 6.5% (vs. 1.4%) to 46.5% (vs. 25.8%). Incidence data were consistent with prevalence findings and showed elevated migraine incidence among people with PTSD, with reported estimates of 48.5% (vs. 39.5%) and 5.74 per 1000 PY(vs. 1.22 per 1000 PY).

### Effect estimates

Most studies (*n* = 6) did not report effect estimates (Table 3). Other studies provided adjusted ORs (*n* = 3), RR (*n* = 1), or HR (*n* = 1), primarily adjusted for demographic variables (e.g., age, sex, income, marital status) as well as multiple physical and psychiatric disorders. All reported effect estimates indicated associations greater than 1, suggesting that PTSD is linked to an increased likelihood of migraine. The highest effect estimate was 3.83 (HR) (95% confidence interval [CI]: 2.82–5.20), whereas the lowest was 1.20 (RR) (95% CI: 1.14–1.27).

**TABLE 3:** Adjusted effect estimates and confounders for the association between PTSD and migraine.

Author(s) + (year)	Effect estimates	Confounders
Barer et al.^28^	N/R	
El‐Gabalawy et al.^39^	N/R	
Friedman et al.^29^	AOR: 2.85 (95% CI: 2.18–3.74)	Age, education, BMI, Mestizo ethnicity, marital status, employment, difficulty paying for the basics or for medical care, parity, planned pregnancy, gestational age, childhood abuse, lifetime intimate partner violence, depression
Friedman et al.^30^	N/R	
Gasperi et al.^31^	N/R	
Herbert et al.^32^	N/R	
Rao et al.^33^	N/R	
Smitherman and Kolvivas^34^	AOR: 1.75 (95% CI: 1.15–2.68)	Sex, anxiety, trauma, PTSD‐symptom severity, depression
Vun et al.^35^	AOR: 2.55 (95% CI: 1.75–3.72)	Age, sex, marital status, rank, Canadian Forces type, education, income, ethnicity, all other mental disorders excluding the mental disorder of interest
Crowe et al.^41^	ARR: 1.20 (95% CI: 1.14–1.27)	Race, education, marital status, high blood pressure, high cholesterol, alcohol intake, smoking, BMI
Huang et al.^42^	HR: 3.83 (95% CI: 2.82–5.20)	Age, sex, level of urbanization, income, depressive disorder, hypertension, dyslipidemia, diabetes mellitus, cerebrovascular diseases, head injury, epilepsy, meningitis, encephalitis

Abbreviations: AOR, adjusted odds ratio; ARR, adjusted risk ratio; BMI, body mass index; CI, confidence interval; HR, hazard ratio; N/R, not reported; PTSD, posttraumatic stress disorder.

## DISCUSSION

In our systematic review with 11 included studies, we observed variations in study quality and methodological approaches. Migraine prevalence and incidence were consistently higher among individuals with PTSD compared to those without. Across nine studies, migraine prevalence in the population with PTSD ranged from 6.5% (vs. 1.4%) to 46.5% (vs. 25.8%). All prevalence and incidence ratios reported exceeded 1.0, with a maximum of 4.7, indicating a robust association across different populations and methodologies. The association was slightly more pronounced in military samples, although high migraine incidence and prevalence estimates among individuals with PTSD were similarly observable in non‐military populations. Adjusted effect estimates reported from the included studies also suggested an increased risk of migraine in individuals with PTSD.

### Methodological heterogeneity and quality assessment

In our quality assessment, we observed substantial variability in methodological quality; for instance, regarding the assessment of PTSD and migraine. Although several studies applied standardized criteria (e.g., ICD, DSM), different versions and adaptations were used. Assessments for both migraine and PTSD varied between clinical interviews and self‐report tools, which, due to lower specificity, may lead to over‐ or underestimation of prevalence and potential misclassification.^43^, ^44^

Further studies, especially those with large sample sizes, relied on claims data. These studies, on the one hand, may also be prone to information bias due to misclassification because coding practices do not precisely reflect underlying clinical conditions. On the other hand, this introduces the risk of selection bias because registered healthcare users are more likely to seek and receive medical care.^45^, ^46^ Furthermore, most of the included studies were cross‐sectional, which limits conclusions about causality.

Another methodological issue was that detailed descriptions of the setting and source population used for participant recruitment were often not provided. Furthermore, the baseline characteristics of the study populations largely differed regarding age, sex distribution, and specific predisposed subgroups (e.g., veterans). This heterogeneity may limit comparability, reduce generalizability, and could also contribute to varying findings for the prevalence and incidence values. However, despite this substantial variability in methodological quality, study designs, data, and assessment tools, all 11 included studies consistently found that migraine prevalence and incidence were higher among individuals with PTSD compared to those without.

### PTSD and migraine prevalence and incidence

Across the included studies, overall PTSD prevalence ranged between 0.5% and 50%, with the majority of the studies exceeding 15%. These figures are substantially higher than the cross‐national prevalence of 3.9% observed in population‐based surveys.^47^ Such a discrepancy is likely due to differences in population characteristics and methodologies. The comparatively high PTSD prevalence observed in our review may reflect the limited number of studies conducted in general population samples. Among the included studies, four focused on military populations,^31^, ^32^, ^35^, ^39^ which are known to report elevated prevalences of PTSD.^48^, ^49^ The prevalence estimates reported in these studies are consistent with those observed in war‐exposed populations. A recent meta‐analysis of 41 surveys conducted among war survivors reported a point prevalence of 26.5% for PTSD,^50^ whereas other studies conducted in the same context reported lifetime prevalences between 7.7% and 17%.^51^, ^52^ Still, non‐military studies reported a higher average PTSD prevalence than military studies, highlighting the relevance of PTSD beyond predisposed populations. Additionally, most of the included studies (*n* = 10) were conducted in high‐income countries,^53^ where PTSD is significantly elevated compared to middle‐ or low‐income countries.^47^ Together, these factors may explain the comparatively high prevalence values, while also reflecting the heterogeneity of epidemiological data on PTSD.

Migraine prevalence was generally more homogenous, with most values below 10%, which is lower than population‐based estimates of 11% to 15% reported in other studies.^1^, ^54^, ^55^ Two studies^29^, ^34^ reported considerably higher prevalences (25.8% and 33.5%), likely reflecting characteristics of the sampled populations, including pregnant women and students. These groups may be particularly vulnerable to migraine because they are associated with hormonal fluctuations^56^, ^57^ and lifestyle‐related factors such as psychosocial stress, irregular sleep, and dietary habits.^58^, ^59^, ^60^ Indeed, the highest estimate was observed among female nurses, with a substantially high incidence of migraine not only in those with PTSD (48.5%) but also in those without PTSD (39.5%),^41^ exceeding figures reported from population‐based studies. Yet, the study did not provide a clear justification for this finding, and reported data lacked clarity due to the presentation of multiple analyses including different subpopulations. Nonetheless, employment within the healthcare sector is a known risk factor for migraine.^61^, ^62^, ^63^ Additionally, all three studies included predominantly female samples (63%–100%), which explains the higher frequencies because migraine is known to be more common in females.^56^, ^64^, ^65^ These findings emphasize the need of sex‐specific analyses and enhanced awareness of hormonal and occupational risk factors in migraine contexts.

### Migraine prevalence and incidence in PTSD

In four studies,^29^, ^33^, ^34^, ^35^ migraine prevalence in PTSD populations exceeded 20%, with a maximum of 46.5%, whereas such elevated estimates were found in only two of the non‐PTSD groups.^29^, ^34^ The magnitude of this difference was substantial, with prevalence ratios of migraine in patients with PTSD versus those without ranging from 1.2 to 4.6. These findings indicate a consistently increased likelihood of migraine in individuals with PTSD, regardless of population or methodology. In line with our findings, data reported by Guilloton et al.^66^ indicated that approximately 55% of individuals with PTSD reported persistent headache. Similarly, a study on Hurricane Katrina survivors demonstrated that PTSD symptoms were associated with an increased risk of frequent headache, including migraine.^67^

Whereas most existing research has focused on the prevalence of PTSD among individuals with migraine, with reported comorbidity rates of 20%–30%,^22^, ^38^, ^68^, ^69^ our review emphasizes the reverse direction. The association appears especially pronounced in people with chronic migraine, which leads to the assumption that PTSD might contribute to headache chronification.^68^ Although at‐risk populations such as veterans may be vulnerable to both disorders, our findings show an equally strong association in general population samples, underscoring the broader relevance of the PTSD‐migraine comorbidity in clinical and public health contexts.

Two included studies provided longitudinal data,^41^, ^42^ which demonstrated that individuals with PTSD had a significantly increased risk of developing migraine over time compared to those without PTSD, with incidence ratios of 1.2 and 4.7, respectively. Peterlin et al. similarly found that 69% of people with migraine reported PTSD before the onset of migraine.^38^ The strong bidirectional association highlights the need to identify and manage both conditions as modifiable risk factors to reduce impairment and prevent chronification. Future research should prioritize longitudinal investigations to clarify the temporal dynamics of their interaction, their trajectories, and to identify potential therapeutic targets along their course.

### Pathophysiological mechanisms linking PTSD and migraine

Evidence on the pathophysiological mechanisms underlying the PTSD‐migraine relationship remains inconsistent because most research focused on chronic pain in patients with PTSD.^70^ One frequently discussed hypothesis involves dysfunction of the hypothalamic–pituitary–adrenal axis. Accordingly, chronic stress may lead to cortisol dysregulation and neuroplastic changes in stress‐ and pain‐related brain regions.^71^, ^72^, ^73^, ^74^ These changes may increase vulnerability to migraine, whereas migraine itself may aggravate stress‐related dysregulation.^73^

Another potential mechanism is central sensitization (CS), clinically defined by widespread pain, sensory hypersensitivity, and intensified pain perception,^75^, ^76^ all of which are associated with PTSD.^77^ Conceptually, CS describes a persistent state of heightened excitability in the central nervous system, triggered by trauma and resulting in exaggerated pain responses even to harmless stimuli.^78^, ^79^ In PTSD, sustained autonomic arousal may change central pain modulation, including enhanced synaptic efficacy, and decreased inhibitory control of nociceptive signaling.^70^, ^79^, ^80^, ^81^ In migraine, CS may promote chronification, particularly through sensitization of trigeminovascular pathways due to altered processing in the trigeminal nucleus caudalis.^82^, ^83^, ^84^, ^85^ PTSD‐related hyperarousal could further reinforce these mechanisms and vice versa. Taken together, these findings support the idea of a bidirectional amplification effect mediated by chronic hyperarousal and CS. Further investigation is needed to improve the understanding of pathways to pain chronification in trauma‐exposed individuals.

Screening for PTSD should therefore be considered, especially in individuals with chronic migraine, who experience a high symptom burden or a reduced response to conventional treatments. Expanding the therapeutic approach to a comprehensive therapy strategy by including psychotherapeutic trauma interventions may improve clinical outcomes and support the development of targeted, interdisciplinary treatment concepts.^86^, ^87^, ^88^ This, in turn, could help prevent chronification, reduce symptom burden, and maintain a high level of quality of life for affected individuals.

### Association of PTSD and migraine compared to mood disorders

Prior research on the association between migraine and psychiatric comorbidities has primarily focused on mood disorders such as anxiety and depression. However, recent evidence from different populations suggests that PTSD is similarly relevant in this context, with prevalence estimates exceeding those of depression.^89^ Supporting these findings, a study^29^ included in our review reported a twofold increased risk for PTSD among individuals with migraine compared to individuals without headache disorders. This association remained significant even after adjusting for depression (OR: 1.97; 95% CI: 1.64–2.37). In line with those findings, Peterlin et al.^23^ demonstrated that migraine was more strongly associated with PTSD than with major depression or anxiety disorders, and that comorbid PTSD increased the risk for chronic migraine beyond the effect of depression alone. These findings suggest that PTSD may have a comparably strong, if not stronger, influence on migraine occurrence and progression than other common psychiatric comorbidities, highlighting its clinical importance and the need for systematic assessment in primary headache care.

## STRENGTHS AND LIMITATIONS

To our knowledge, this is the first systematic review specifically examining the prevalence and incidence of migraine among individuals with and without PTSD. A major strength of this review was its comprehensive search strategy, which applied no restrictions on publication language or date and resulted in the screening of approximately 13,000 records. To enhance coverage, we performed a forward and backward citation analysis and assessed 236 full‐text articles, which enabled us to include studies that did not explicitly focus on the association of PTSD and migraine but reported relevant data in the full text.

However, some important limitations should be considered. First, potentially relevant studies may have been missed because they did not indicate the association of interest in their title or abstract. Although we addressed this through a broad search strategy and citation analysis in Web of Science, not all references were accessible, potentially limiting the comprehensiveness of our analysis. Second, because most included studies did not primarily focus on the PTSD‐migraine association but still provided prevalence or incidence data, manual calculation of estimates was necessary, and adjusted findings were not available. Third, data extraction was done by only one reviewer but cross‐checked and recalculated by a second reviewer. However, this was done with knowledge of the numbers obtained by the initial reviewer and therefore was not fully independent, possibly leading to errors or bias. Fourth, because the included studies were very heterogenous, a meta‐analysis pooling all data was not possible. However, this was already anticipated when planning this review and included in the protocol accordingly.

## CONCLUSION

This systematic review provides substantial evidence for a strong association between PTSD and migraine, reflected in consistently elevated prevalence estimates among individuals with PTSD and further supported by incidence data indicating an increased risk over time. The strength of the association was robust across diverse populations, demonstrating its clinical relevance, even though the interpretation of this relationship should consider the methodological heterogeneity across studies. Therefore, future research should employ the use of standardized diagnostic criteria and transparent reporting to enhance data comparability and provide more reliable evidence.

Nevertheless, our findings highlight the need to systematically assess trauma exposure with validated diagnostic tools in patients with primary headache disorders, especially migraine. PTSD screening, initiating a more comprehensive therapeutic approach, should be considered particularly in individuals with migraine who insufficiently respond to conventional treatment to prevent chronification and improve long‐term outcomes.

## AUTHOR CONTRIBUTIONS

**Lucie Nitsche:** Conceptualization; investigation; writing – original draft; methodology; visualization; formal analysis; data curation. **Jasmin Helbach:** Conceptualization; investigation; methodology; validation; writing – review and editing; software. **Sarah Stubenrauch:** Investigation; validation. **Meret Lakeberg:** Investigation; validation. **Carsten Bantel:** Conceptualization; writing – review and editing; validation; methodology; supervision; project administration. **Falk Hoffmann:** Conceptualization; methodology; validation; writing – review and editing; project administration; supervision.

## FUNDING INFORMATION

This research did not receive any specific grant from funding agencies in the public, commercial, or not‐for‐profit sectors.

## CONFLICT OF INTEREST STATEMENT

**Lucie Nitsche, Jasmin Helbach, Sarah Stubenrauch, Meret Lakeberg, Carsten Bantel**, and **Falk Hoffmann** declare that there are no conflicts of interest.

## Supporting information


**Data S1:** Supplementary Information.

## Data Availability

All data supporting the findings of this study are available in both the article and the supplementary file.
