# Supplementary material for: The association between posttraumatic stress disorder and migraine: A systematic review
Source: Headache. 2026 Feb 18;66(4):963–75. doi: 10.1111/head.70058 (PMC13044569; doi:10.1111/head.70058)
Supplement: Supplementary file 1 — Data S1: Supplementary Information. [file HEAD-66-963-s001.pdf]

Table S1 – Search Strategy

PubMed (Searched on November 22, 2024)

**PTSD**	**Matches**
"stress disorders, post traumatic"[MeSH Terms] OR "PTSD"[Title/Abstract] OR "post-traumatic stress disorder"[Title/Abstract] OR "posttraumatic stress disorder"[Title/Abstract] OR "post-traumatic syndrome" [Title/Abstract] OR "posttraumatic syndrome" [Title/Abstract] OR "post-traumatic stress" [Title/Abstract] OR "posttraumatic stress"[Title/Abstract] OR "traumatic stress disorder"[Title/Abstract] OR "traumatic stress"[Title/Abstract]	67,891
**Migraine**	
"Migraine Disorders"[MeSH Terms] OR "migrain*"[Title/Abstract]	50,154
**PTSD vs. Non-PTSD**	
"without PTSD"[Title/Abstract:~1] OR "without post traumatic stress disorder"[Title/Abstract:~1] OR "without posttraumatic stress disorder"[Title/Abstract:~1] OR "non PTSD" [Title/Abstract] OR "non-PTSD" [Title/Abstract] OR "non post traumatic stress disorder" [Title/Abstract] OR "non posttraumatic stress disorder" [Title/Abstract] OR "control group"[Title/Abstract] OR "controls" [Title/Abstract]	1,502,768
**Migraine OR PTSD vs. Non-PTSD**	
("Migraine Disorders"[MeSH Terms] OR "migrain*"[Title/Abstract]) OR ("without PTSD"[Title/Abstract:~1] OR "without post traumatic stress disorder"[Title/Abstract:~1] OR "without posttraumatic stress disorder"[Title/Abstract:~1] OR "non PTSD" [Title/Abstract] OR "non-PTSD" [Title/Abstract] OR "non post traumatic stress disorder" [Title/Abstract] OR "non posttraumatic stress disorder" [Title/Abstract] OR "control group"[Title/Abstract] OR "controls" [Title/Abstract])	1,547,942
**Total**	
("stress disorders, post traumatic"[MeSH Terms] OR "PTSD"[Title/Abstract] OR "post-traumatic stress disorder"[Title/Abstract] OR "posttraumatic stress disorder"[Title/Abstract] OR "post-traumatic syndrome" [Title/Abstract] OR "posttraumatic syndrome" [Title/Abstract] OR "post-traumatic stress" [Title/Abstract] OR "posttraumatic stress"[Title/Abstract] OR "traumatic stress disorder"[Title/Abstract] OR "traumatic stress disorders"[Title/Abstract] OR "traumatic stress"[Title/Abstract]) AND (("Migraine Disorders"[MeSH Terms] OR "migrain*"[Title/Abstract]) OR ("without PTSD"[Title/Abstract:~1] OR "without post traumatic stress disorder"[Title/Abstract:~1] OR "without posttraumatic stress disorder"[Title/Abstract:~1] OR "non PTSD" [Title/Abstract] OR "non-PTSD" [Title/Abstract] OR "non post traumatic stress disorder" [Title/Abstract] OR "non posttraumatic stress disorder" [Title/Abstract] OR "control group"[Title/Abstract] OR "controls" [Title/Abstract]))	6,710

PsycInfo (Searched on November 22, 2024)

PTSD	Matches
MM "Posttraumatic Stress Disorder" OR TI ("PTSD" OR "post-traumatic stress disorder" OR "posttraumatic stress disorder" OR "post-traumatic syndrome" OR "posttraumatic syndrome" OR "post-traumatic stress" OR "posttraumatic stress" OR "traumatic stress disorder" OR "traumatic stress") OR AB ("PTSD" OR "post-traumatic stress disorder" OR "posttraumatic stress disorder" OR "post-traumatic syndrome" OR "posttraumatic syndrome" OR "post-traumatic stress" OR "posttraumatic stress" OR "traumatic stress disorder" OR "traumatic stress")	63,479
**Migraine**	
MM "Migraine Headache" OR TI "migrain*" OR AB "migrain*"	13,977
**PTSD vs. Non-PTSD**	
TI ("without N1 PTSD" OR "without N1 post traumatic stress disorder" OR "without N1 posttraumatic stress disorder" OR "non PTSD" OR "non-PTSD" OR "non post traumatic stress disorder" OR "non posttraumatic stress disorder" OR "control group" OR "controls") OR AB ("without N1 PTSD" OR "without N1 post traumatic stress disorder" OR "without N1 posttraumatic stress disorder" OR "non PTSD" OR "non-PTSD" OR "non post traumatic stress disorder" OR "non posttraumatic stress disorder" OR "control group" OR "controls")	255,320
**Migraine OR PTSD vs. Non-PTSD**	
(MM "Migraine Headache" OR TI "migrain*" OR AB "migrain*") OR (TI ("without N1 PTSD" OR "without N1 post traumatic stress disorder" OR "without N1 posttraumatic stress disorder" OR "non PTSD" OR "non-PTSD" OR "non post traumatic stress disorder" OR "non posttraumatic stress disorder" OR "control group" OR "controls") OR AB ("without N1 PTSD" OR "without N1 post traumatic stress disorder" OR "without N1 posttraumatic stress disorder" OR "non PTSD" OR "non-PTSD" OR "non post traumatic stress disorder" OR "non posttraumatic stress disorder" OR "control group" OR "controls"))	267,256
**Total**	
(MM "Posttraumatic Stress Disorder" OR TI ("PTSD" OR "post-traumatic stress disorder" OR "posttraumatic stress disorder" OR "post-traumatic syndrome" OR "posttraumatic syndrome" OR "post-traumatic stress" OR "posttraumatic stress" OR "traumatic stress disorder" OR "traumatic stress") OR AB ("PTSD" OR "post-traumatic stress disorder" OR "posttraumatic stress disorder" OR "post-traumatic syndrome" OR "posttraumatic syndrome" OR "post-traumatic stress" OR "posttraumatic stress" OR "traumatic stress disorder" OR "traumatic stress")) AND ((MM "Migraine Headache" OR TI "migrain*" OR AB "migrain*") OR (TI ("without N1 PTSD" OR "without N1 post traumatic stress disorder" OR "without N1 posttraumatic stress disorder" OR "non PTSD" OR "non-PTSD" OR "non post traumatic stress disorder" OR "non posttraumatic stress disorder" OR "control group" OR "controls") OR AB ("without N1 PTSD" OR "without N1 post traumatic stress disorder" OR "without N1 posttraumatic stress disorder" OR "non PTSD" OR "non-PTSD" OR "non post traumatic stress disorder" OR "non posttraumatic stress disorder" OR "control group" OR "controls")))	4372

Embase (Searched on November 22, 2024)

PTSD	Matches
"posttraumatic stress disorder"/exp OR "PTSD":ti,ab OR "post-traumatic stress disorder":ti,ab OR "posttraumatic stress disorder":ti,ab OR "post-traumatic syndrome":ti,ab OR "posttraumatic syndrome":ti,ab OR "post-traumatic stress":ti,ab OR "posttraumatic stress":ti,ab OR "traumatic stress disorder":ti,ab OR "traumatic stress":ti,ab	99,278
**Migraine**	
"Migraine"/exp OR "migrain*":ti,ab	94,122
**PTSD vs. Non-PTSD**	
"without NEAR/1 PTSD":ti,ab OR "without NEAR/1 post traumatic stress disorder":ti,ab OR "without NEAR/1 posttraumatic stress disorder":ti,ab OR "non PTSD":ti,ab OR "non-PTSD":ti,ab OR "non post traumatic stress disorder":ti,ab OR "non posttraumatic stress disorder":ti,ab OR "control group":ti,ab OR "controls":ti,ab	2,113,072
**Migraine OR PTSD vs. Non-PTSD**	
("Migraine"/exp OR "migrain*":ti,ab) OR ("without NEAR/1 PTSD":ti,ab OR "without NEAR/1 post traumatic stress disorder":ti,ab OR "without NEAR/1 posttraumatic stress disorder":ti,ab OR "non PTSD":ti,ab OR "non-PTSD":ti,ab OR "non post traumatic stress disorder":ti,ab OR "non posttraumatic stress disorder":ti,ab OR "control group":ti,ab OR "controls":ti,ab)	2,199,004
**Total**	
("posttraumatic stress disorder"/exp OR "PTSD":ti,ab OR "post-traumatic stress disorder":ti,ab OR "posttraumatic stress disorder":ti,ab OR "post-traumatic syndrome":ti,ab OR "posttraumatic syndrome":ti,ab OR "post-traumatic stress":ti,ab OR "posttraumatic stress":ti,ab OR "traumatic stress disorder":ti,ab OR "traumatic stress disorders":ti,ab OR "traumatic stress":ti,ab) AND (("Migraine Disorders"/exp OR "migrain*":ti,ab) OR ("without NEAR/1 PTSD":ti,ab OR "without NEAR/1 post traumatic stress disorder":ti,ab OR "without NEAR/1 posttraumatic stress disorder":ti,ab OR "non PTSD":ti,ab OR "non-PTSD":ti,ab OR "non post traumatic stress disorder":ti,ab OR "non posttraumatic stress disorder":ti,ab OR "control group":ti,ab OR "controls":ti,ab))	8475

Table S2 – Quality assessment for cross-sectional studies.

	Criteria	VUN[1]	SMITHERMAN[2]	RAO[3]	HERBERT[4]	GASPERI[5]	FRIEDMAN[6]	FRIEDMAN[7]	EL-GABALAWY[8]	BARER[9]
**1. Were the criteria for inclusion in the sample clearly defined?**	**Yes**: It was clearly defined which inclusion and exclusion criteria were used to identify participants. e.g. answered a screening question in a survey; completed data on pre-defined information (e.g. outcome variable) at a pre-defined timepoint (e.g. baseline), only include a subgroup (e.g., only females or a specified age group). **No**: In the absence of a description of the methodology used to derive the sample from the source population and the composition of the sample, the study is considered incomplete.	yes	yes	yes	yes	yes	yes	yes	no	yes
**2. Were the study subjects and the setting described in detail?**	**Yes**: The authors provide a clear description of the population from which the study participants were selected or recruited, including demographics, location, and time period. **No**: The authors provide no description of the demographics, location, or time period of the population from which the study participants were selected or recruited. It is insufficient to refer to another study in which the population is described in more detail.	no	no	yes	no	yes	no	no	no	no
**3. Was the exposure measured in a valid and reliable way?**	**Yes**: PTSD was diagnosed based on established clinical diagnostic criteria (i.e. DSM or ICD), self-reports (i.e. PTSD scale), or claims data. **No**: It was not described how PTSD was diagnosed, or the diagnostic measures did not appear to be valid (self-reported data, collected without the use of standardized instruments). If a valid instrument was used, but it was not explained which/how many items of the instrument had to be fulfilled to diagnose PTSD, we rated this as no.	no	yes	no	no	yes	yes	yes	yes	yes
**4. Were objective, standard criteria used for measurement of the condition?**	**Yes**: It clearly describes what the underlying condition is and how it has been defined. **No:** No underlying condition specified or defined. If the condition is defined as trauma, but there is no specification of what is meant by trauma, we rated this as no.	yes	yes	no	no	yes	not applicable	no	yes	no
**5. Were the outcomes measured in a valid and reliable way?**	**Yes**: Migraine was diagnosed based on established clinical diagnostic criteria (i.e. DSM or ICD), self-reports (i.e.ID-Migraine screener), or claims data. **No**: It was not described how migraine was diagnosed, or the diagnostic measures did not appear to be valid (self-reported data, collected without the use of standardized instruments). If a valid instrument was used, but it was not explained which/how many items of the instrument had to be fulfilled to diagnose migraine, we rated this as no.	no	yes	yes	no	no	yes	yes	no	yes

Table S3 – Quality assessment for cohort studies.

	Criteria	CROWE[10]	HUANG[11]
1. **Were the two groups similar and recruited from the same population?**	**Yes**: Participant characteristics were described for both groups (PTSD and no-PTSD), and it was determined that participants within and between groups exhibited similar characteristics in relation to exposure (e.g. age, risk factors being studied). In addition, the inclusion and exclusion criteria used to identify participants were clearly defined.<br>**No**: The participant characteristics of the two groups were not sufficiently detailed to ensure equivalent exposure characteristics within and between groups. In the absence of a description of the methodology used to derive the sample from the source population and to build the two groups, the study is considered incomplete.	yes	yes
2. **Were the exposures measured similarly to assign people to both exposed and unexposed groups?**	**Yes**: The allocation of participants to the PTSD or no-PTSD group was executed following the same set of diagnostic criteria for all participants.<br>**No**: The allocation of participants to the PTSD or no-PTSD group was executed following different sets of diagnostic criteria.	yes	yes
3. **Was the exposure measured in a valid and reliable way?**	**Yes**: PTSD was diagnosed based on established clinical diagnostic criteria (i.e. DSM or ICD), self-reports (i.e. PTSD scale), or claims data.<br>**No**: It was not described how PTSD was diagnosed, or the diagnostic measures did not appear to be valid (self-reported data, collected without the use of standardized instruments). If a valid instrument was used, but it was not explained which/how many items of the instrument had to be fulfilled to diagnose PTSD, we rated this as no.	yes	yes
4. **Were the groups/participants free of the outcome at the start of the study (or at the moment of exposure)?**	**Yes**: The methods section should include a statement that all participants from both groups (PTSD and no-PTSD) were free of the outcome of interest (migraine) at the start of the study.<br>**No**: Not all participants in both groups (PTSD and no-PTSD) were free of the outcome of interest (migraine) at the start of the study	yes	yes
5. **Were the outcomes measured in a valid and reliable way?**	**Yes**: Migraine was diagnosed based on established clinical diagnostic criteria (i.e. DSM or ICD), self-reports (i.e. ID-Migraine screener), or claims data.<br>**No**: It was not described how migraine was diagnosed, or the diagnostic measures did not appear to be valid (self-reported data, collected without the use of standardized instruments). If a valid instrument was used, but it was not explained which/how many items of the instrument had to be fulfilled to diagnose migraine, we rated this as no.	no	yes
6. **Was the follow up time reported and sufficient to be long enough for outcomes to occur?**	**Yes**: The follow-up period lasted a minimum of 24 months.<br>**No**: The follow-up period was less than 24 months.	yes	yes
7. **Was follow up complete, and if not, were the reasons to loss to follow up described and explored?**	**Yes**: All participants considered, response >80%, with the reasons for loss to follow-up explored and described.<br>**No**: No exploration or description of reasons for loss to follow-up, or response rate <80%.	no	not applicable
8. **Were strategies to address incomplete follow up utilized?**	**Yes**: The loss to follow-up was managed appropriately and was considered in the analysis.<br>**No**: No indication that the loss to follow up was managed appropriately or was considered in the analysis.	no	not applicable

S4: Calculations:

Barer[9]:

- Overall Migraine Prevalence: (368+570)/16,672 = 5.6% (cf. Tab. 1)

El-Gabalawy[8]:

Explanation: The subgroup totals do not match the stated overall sample size (n = 3,157). For our calculations, we used the combined sample size derived from subgroup data (289 partial PTSD + 182 full PTSD + 2,078 trauma exposed + 384 not trauma exposed = 2,933), as this reflects participants with available PTSD and migraine data.

- Overall PTSD-Prevalence: (289+182)/2,933= 16.1% (cf. Tab. 2)
- Overall Migraine Prevalence: (11+109+27+39)/2,933 = 6.3% (cf. Tab. 2)
- Migraine Prevalence in PTSD: (27+39)/(289+182) =14.0% (cf. Tab. 2)
- Migraine Prevalence in non-PTSD: (11+109)/(348+2,078) = 4.9% (cf. Tab. 2)

Friedman[6]:

- Overall Migraine Prevalence: (613+366)/2,922= 33.5% (cf. Tab. 1)
- Migraine Prevalence in PTSD: (276+232)/1,093 = 46.5% (cf. Tab. 1)
- Migraine Prevalence in non-PTSD: (979-508)/(2,922-1,093) = 25.8% (cf. Tab. 1)

Friedman[7]:

- Overall Migraine Prevalence: 2,190,305/156,172,826 = 1.4% (cf. Tab. 1)
- Migraine Prevalence in PTSD: 54,429/840,338 = 6.5% (cf. Tab. 1)
- Migraine Prevalence in non-PTSD: (2,190,305-54,429)/(156,172,826-840,338) = 1.4% (cf. Tab. 1)

Gasperi[5]:

Explanation: The subgroup totals do not match the stated overall sample size (n = 4,680). For our calculations, we used the combined sample size derived from subgroup data (908 PTSD + 3,646 non-PTSD = 4,554), as this reflects participants with available PTSD and migraine data.

- Overall PTSD Prevalence: 908/4,554 = 19.9% (cf. Tab. 1)
- Overall Migraine Prevalence: ((908×0.173)+(3,646×0.052))/4,554 = 347/4,554 = 7.6% (cf. Tab. 1).
- Migraine Prevalence in PTSD : 0.173x908 = 157 (cf. Tab. 1)
- Migraine prevalence in non-PTSD: 0.052x3,646 = 190 (cf. Tab. 1)

Herbert[4]:

- Migraine Prevalence in PTSD: (6,727+4,243)/56,461 = 19.4% (cf. Tab. 1)
- Migraine Prevalence in non-PTSD: ((22,252+7,828) – (6,727+4,243))/ (338,217-56,461) = 6.8% (cf. Tab. 1)

Rao[3]:

- Proportion female: (2,216+178+188+55)/(4,535+244+236+68) = 51.9% (cf. Tab 1)
- Overall PTSD-Prevalence: (244+68)/5,083 = 6.1% (cf. Tab. 1)
- Overall Migraine Prevalence: (236+68)/5,083 = 6.0% (cf. Tab. 1)
- Migraine Prevalence in PTSD: 68/ (68+244) = 21.8% (cf. Tab. 1)
- Migraine Prevalence in non-PTSD: 236/(236+4,535) = 4.9% (cf. Tab. 1)

Smitherman[2]:

- Overall PTSD-Prevalence: (77+107)/1,051= 17.5% (cf. Tab. 2)
- Migraine Prevalence in PTSD: 77/(77+107) = 41.8% (cf. Tab. 2)
- Migraine Prevalence in non-PTSD: (300-77)/ (1,051-184) = 25.7% (cf. Tab. 1)

Vun[1]:

- Migraine Prevalence in PTSD: 86/348 = 24.7% (cf. Tab. 2)
- Migraine Prevalence in non-PTSD: 527/(6,696-348) = 8.3% (cf. Tab. 1 and 2)

Crowe[10]:
Explanation: For the calculations, only the subgroup reporting migraine incidence among participants with PTSD (1,105+1,174 = 2,279) + without PTSD ((8,076+12,513 trauma exposed) + (2,412 + 3,535 no trauma) = 26,536), n = 28,815 was used, not the full study cohort (n = 33,327).

- Overall PTSD Prevalence: (1,105+1,174)/28,815 = 7.9% (cf. Tab. 4)
- Overall Migraine Prevalence: (1,105+8,076+2,412)/28,815 = 40.2% (cf. Tab.4)
- Migraine Incidence in non-PTSD: (8,076+2,412)/((8,076+2,412)+(12,513+3,535)) = 39.5% (cf. Tab. 4)

Huang[11]:
- Overall PTSD Prevalence: 5,664/28,220 = 20.0% (cf. Tab. 1)
- Overall Migraine Incidence: (5.7×5,644+1.2×22,576)/(5,644+22,576)=2.10 per 1,000 PY (cf. Tab. 1)

Table S5 – Excluded articles with reason for exclusion

Article	Reason for exclusion
Abramovitz, L. M., et al. (2021). "Posttraumatic Stress Disorder in a Cohort of Pregnant Active Duty U.S. Military Servicewomen." Journal of Traumatic Stress 34(3): 586-595.	No Data on Migraine
Abrams, T. E., et al. (2013). "Patterns of illness explaining the associations between posttraumatic stress disorder and the use of CT." Radiology 267(2): 470-478.	No Data on Migraine
Adams, R. S., et al. (2019). "Postdeployment Polytrauma Diagnoses Among Soldiers and Veterans Using the Veterans Health Affairs Polytrauma System of Care and Receipt of Opioids, Nonpharmacologic, and Mental Health Treatments." Journal of Head Trauma Rehabilitation 34(3): 167-175.	No comparison of PTSD vs. non-PTSD
Afari, N., et al. (2009). "PTSD, Combat injury, and headache in Veterans Returning from Iraq/Afghanistan." Headache 49(9): 1267-1276.	No comparison of PTSD vs. non-PTSD
Agibalova, T. V., et al. (2014). "Features of the Formation, Course, and Treatment of Alcohol Dependence in Patients with Post-Traumatic Stress Disorder." Neuroscience and Behavioral Physiology 44(9): 1068-1072.	No comparison of PTSD vs. non-PTSD
Ahmadi, N., et al. (2013). "Post-Traumatic Stress Disorder Is Associated with Increased Incidence of Insulin Resistance and Metabolic Syndrome." Journal of the American College of Cardiology 61(10).	Review, Editorial, PhD Thesis, Conference Abstract
Akbulut-Yuksel, M., et al. (2024). "Untold Story of Wartime Children: Results of the Vietnam Health and Aging Study." Population Research and Policy Review 43(2).	No Data on Migraine
Al-Hamzawi, A. O., et al. (2014). "The role of common mental and physical disorders in days out of role in the Iraqi general population: results from the WHO World Mental Health Surveys." Journal of Psychiatric Research 53: 23-29.	No comparison of PTSD vs. non-PTSD
Al-Saffar, S., et al. (2002). "Long-term consequences of unrecognised PTSD in general outpatient psychiatry." Social Psychiatry and Psychiatric Epidemiology 37(12): 580-585.	No Data on Migraine
Alemi, F., et al. (2017). "Citalopram is less effective for patients with neurological disorder and/or post-traumatic stress disorder." Personalized Medicine in Psychiatry 4-6: 32-38.	No Data on Migraine
Alfano, D. P., et al. (2000). "Post-traumatic stress disorder and mild traumatic brain injury." Brain and Cognition 44(1): 90-94.	Sample size (<100 general; <50 PTSD; <50 non-PTSD)
Amital, D., et al. (2006). "Posttraumatic stress disorder, tenderness, and fibromyalgia syndrome: are they different entities?" Journal of Psychosomatic Research 61(5): 663-669.	No Data on Migraine
Andersen, J., et al. (2010). "Association between posttraumatic stress disorder and primary care provider-diagnosed disease among Iraq and Afghanistan veterans." Psychosomatic Medicine 72(5): 498-504.	No Data on Migraine
Andersen, T. E., et al. (2014). "Chronic pain patients with possible co-morbid post-traumatic stress disorder admitted to multidisciplinary pain rehabilitation-a 1-year cohort study." European Journal of Psychotraumatology (5).	No Data on Migraine
Andreski, P., et al. (1998). "Post-traumatic stress disorder and somatization symptoms: a prospective study." Psychiatry Research 79(2): 131-138.	No Data on Migraine
Anthony, S. E., et al. (2023). "Suicide in Veterans with Post-Traumatic Headache: An Analysis of Health Systems Data." Neurology 100(17).	Review, Editorial, PhD Thesis, Conference Abstract

Arcaya, M. C., et al. (2017). "Association of posttraumatic stress disorder symptoms with migraine and headache after a natural disaster." Health Psychology 36(5): 411-418.	No comparison of PTSD vs. no-PTSD
Asif, A., et al. (2013). "Effect of posttraumatic stress disorder on sleep architecture of patients with obstructive sleep apnea." Sleep 36: A154-A155.	Review, Editorial, PhD Thesis, Conference Abstract
Assefa, M. T., et al. (2024). "A Multipronged Approach to Caring for Women Veterans With Military Environmental Exposures." Women's Health Issues 34(4): 325-330.	Review, Editorial, PhD Thesis, Conference Abstract
Avidor, S., et al. (2016). "Subjective Age and Health in Later Life: The Role of Posttraumatic Symptoms." Journals of Gerontology. Series B: Psychological Sciences and Social Sciences 71(3): 415-424.	No Data on Migraine
Avidor, S., et al. (2021). "What Predicts Unremitting Suicidal Ideation? A Prospective Examination of the Role of Subjective Age in Suicidal Ideation Among Ex-Prisoners of War." Psychological Trauma: Theory, Research, Practice and Policy 13(3): 338-348.	No Data on Migraine
Babakhanyan, I., et al. (2024). "Gender Disparities in Neurobehavioral Symptoms and the Role of Post-Traumatic Symptoms in US Service Members Following Mild Traumatic Brain Injury." Journal of Neurotrauma 41(13-14): e1687-e1696.	No Data on Migraine
Babić, D., et al. (2003). "Comorbidity of the posttraumatic stress disorder and the depression in ex-prisoners of war." Psychiatria Danubina 15(3-4): 195-200.	No Data on Migraine
Baecke, M., et al. (2009). "Cognitive function after pre-eclampsia: an explorative study." Journal of Psychosomatic Obstetrics and Gynaecology 30(1): 58-64.	No comparison of PTSD vs. non-PTSD
Bainomugisa, C. K., et al. (2025). "Shared genetic risk and causal associations between Post-traumatic stress disorder and migraine with antithrombotic agents and other medications." Neurobiol Stress 34: 100703.	No comparison of PTSD vs. non-PTSD
Baird, T., et al. (2018). "Sleep Disturbances in Australian Vietnam Veterans With and Without Posttraumatic Stress Disorder." Journal of Clinical Sleep Medicine 14(5): 745-752.	No Data on Migraine
Balaban, H., et al. (2012). "Migraine prevalence, alexithymia, and post-traumatic stress disorder among medical students in Turkey." Journal of Headache and Pain 13(6): 459-467.	Sample size (<100 general; <50 PTSD; <50 non-PTSD)
Balba, N. M., et al. (2018). "Increased Sleep Disturbances and Pain in Veterans With Comorbid Traumatic Brain Injury and Posttraumatic Stress Disorder." Journal of Clinical Sleep Medicine 14(11): 1865-1878.	No Data on Migraine
Balba, N. M., et al. (2022). "Photosensitivity Is Associated with Chronic Pain following Traumatic Brain Injury." Journal of Neurotrauma 39(17-18): 1183-1194.	No comparison of PTSD vs. non-PTSD
Balba, N., et al. (2020). "Photosensitivity as a marker for chronic pain in patients with TBI and PTSD." Global Advances In Health and Medicine 9: 47-48.	No comparison of PTSD vs. non-PTSD
Barer, Y., et al. (2021). "Post-Traumatic Stress Disorder and risk of Parkinson's Disease- 20 Years Follow-up real world data analysis." Movement Disorder 36(SUPPL 1): S68-S69.	Review, Editorial, PhD Thesis, Conference Abstract
Barrett, D. H., et al. (2002). "Posttraumatic stress disorder and self-reported physical health status among U.S. Military personnel serving during the Gulf War period: a population-based study." Psychosomatics 43(3): 195-205.	No Data on Migraine
Basso, L., et al. (2022). "Sex Differences in Comorbidity Combinations in the Swedish Population." Biomolecules 12(7).	No comparison of PTSD vs. non-PTSD

Bassuk, E. L., et al. (2001). "Post-traumatic stress disorder in extremely poor women: implications for health care clinicians." J Am Med Womens Assoc (1972) 56(2): 79-85.	No Data on Migraine
Bawaadam, H., et al. (2013). "Effect of Gastroesophageal Reflux Disorder on Sleep Architecture in Patients With Obstructive Sleep Apnea." Chest 144(4).	Review, Editorial, PhD Thesis, Conference Abstract
Beckham, J. C., et al. (1997). "Prevalence and correlates of heavy smoking in Vietnam veterans with chronic posttraumatic stress disorder." Addictive Behaviors 22(5): 637-647.	No Data on Migraine
Beckham, J. C., et al. (1998). "Health status, somatization, and severity of posttraumatic stress disorder in Vietnam combat veterans with posttraumatic stress disorder." American Journal of Psychiatry 155(11): 1565-1569.	No Data on Migraine
Beckham, J. C., et al. (2003). "Ambulatory monitoring and physical health report in Vietnam veterans with and without chronic posttraumatic stress disorder." Journal of Traumatic Stress 16(4): 329-335.	No comparison of PTSD vs. non-PTSD
Belding, J. N., et al. (2021). "Occupational Risk of Low-Level Blast Exposure and TBI-Related Medical Diagnoses: A Population-Based Epidemiological Investigation (2005-2015)." International Journal of Environmental Research and Public Health 18(24).	No comparison of PTSD vs. non-PTSD
Berghoff, C. R., et al. (2018). "Psychological flexibility moderates the relation between PTSD symptoms and daily pain interference." Personality and Individual Differences 124: 130-134.	No comparison of PTSD vs. non-PTSD
Beristianos, M. H., et al. (2016). "PTSD and Risk of Incident Cardiovascular Disease in Aging Veterans." American Journal of Geriatric Psychiatry 24(3): 192-200.	No Data on Migraine
Bilevicius, E., et al. (2018). "Posttraumatic stress disorder and chronic pain are associated with opioid use disorder: Results from a 2012-2013 American nationally representative survey." Drug and Alcohol Dependence 188: 119-125.	No Data on Migraine
Bilić, M., et al. (2013). "Quality and Intensity of Low Back Pain in Cronic PTSD Patients." Collegium Antropologicum 37(4): 1229-1236.	No Data on Migraine
Blanchard, M. S., et al. (2006). "Chronic multisymptom illness complex in Gulf War I veterans 10 years later." American Journal of Epidemiology 163(1): 66-75.	No comparison of PTSD vs. non-PTSD
Bomeya, J., et al. (2019). "Associations between neuropsychiatric and health status outcomes in individuals with probable mTBI." Psychiatry Research 272: 531-539.	No Data on Migraine
Brackbill, R. M., et al. (2014). "Chronic physical health consequences of being injured during the terrorist attacks on World Trade Center on September 11, 2001." American Journal of Epidemiology 179(9): 1076-1085.	No Data on Migraine
Braš, M., et al. (2011). "T404 Chronic Pain in Posttraumatic Stress Disorder: An Underestimated Phenomenon." European Journal of Pain Supplements 5(S1): 66-66.	Review, Editorial, PhD Thesis, Conference Abstract
Braš, M., et al. (2019). "The Role of PTSD in Perception of Health-Related Quality of Life and Social Support among Croatian War Veterans." Psychiatr Danub 31(Suppl 5): 761-768.	No Data on Migraine
Breslau, N., et al. (2004). "Sleep in Lifetime Posttraumatic Stress Disorder: a community-based polysomnographic study." Archives of General Psychiatry 61(5): 508-516.	No Data on Migraine
Britvic, D., et al. (2015). "Comorbidities with Posttraumatic Stress Disorder (PTSD) among combat veterans: 15 years postwar analysis." International Journal of Clinical and Health Psychology 15(2): 81-92.	No Data on Migraine

Bromet, E. J., et al. (2002). "Somatic symptoms in Women 11 Years after the Chornobyl Accident: Prevalence and Risk Factors." Environmental Health Perspectives 110 Suppl 4(Suppl 4): 625-629.	No comparison of PTSD vs. non-PTSD
Bryan, J. L., et al. (2021). "Cannabis use disorder and post-traumatic stress disorder: The prevalence of comorbidity in veterans of recent conflicts." Journal of Substance Abuse Treatment 122: 108254.	No Data on Migraine
Bryant, R. A. and A. G. Harvey (1999). "Postconcussive symptoms and posttraumatic stress disorder after mild traumatic brain injury." Journal of Nervous and Mental Disease 187(5): 302-305.	Sample size (<100 general; <50 PTSD; <50 non-PTSD)
Buchwald, D., et al. (2005). "Relationship Between Post-traumatic Stress Disorder and Pain in Two American Indian Tribes." Pain Medicine 6(1): 72-79.	No Data on Migraine
Calhoun, P. S., et al. (2002). "Medical service utilization by veterans seeking help for posttraumatic stress disorder." American Journal of Psychiatry 159(12): 2081-2086.	No Data on Migraine
Carleton, R. N., et al. (2018). "Trauma, Pain, and Psychological Distress." Journal of Psychophysiology 32(2): 75-84.	Sample size (<100 general; <50 PTSD; <50 non-PTSD)
Carlson, J. G., et al. (1994). "Characteristics of veterans in Hawaii with and without diagnoses of post-traumatic stress disorder." Hawaii Medical Journal 53(11): 314-318.	Sample size (<100 general; <50 PTSD; <50 non-PTSD)
Carlson, K. F., et al. (2013). "Headache diagnoses among Iraq and Afghanistan war veterans enrolled in VA: a gender comparison." Headache 53(10): 1573-1582.	No comparison of PTSD vs. non-PTSD
Carmassi, C., et al. (2020). "Disrupted Rhythmicity and Vegetative Functions Relate to PTSD and Gender in Earthquake Survivors." Front Psychiatry 11: 492006.	No Data on Migraine
Carmassi, C., et al. (2021). "Do somatic symptoms relate to PTSD and gender after earthquake exposure? A cross-sectional study on young adult survivors in Italy." CNS Spectr 26(3): 268-274.	No Data on Migraine
Cesur, R., et al. (2015). "Combat exposure and migraine headache: evidence from exogenous deployment assignment." Economics and Human Biology 16: 81-99.	No comparison of PTSD vs. non-PTSD
Chaix, B., et al. (2020). "Psychological distress during the COVID-19 pandemic in France: a national assessment of at-risk populations." Gen Psychiatr 33(6): e100349.	No comparison of PTSD vs. non-PTSD
Chan, L. H. T. and Y. Woldeamanuel (2020). "Clinical predictors associated with persistent versus acute post-traumatic headache." Neurology 94(15).	Review, Editorial, PhD Thesis, Conference Abstract
Chan, T. L. H. and Y. W. Woldeamanuel (2020). "Exploring naturally occurring clinical subgroups of post-traumatic headache." Journal of Headache and Pain 21(1): 12.	No comparison of PTSD vs. non-PTSD
Chemtob, C. M., et al. (1998). "Head injury and combat-related posttraumatic stress disorder." Journal of Nervous and Mental Disease 186(11): 701-708.	No Data on Migraine
Chen, M. H., et al. (2015). "Risk of stroke among patients with post-traumatic stress disorder: Nationwide longitudinal study." British Journal of Psychiatry 206(4): 302-307.	No Data on Migraine
Chen, Y. H., et al. (2017). "Risk of Epilepsy in Individuals With Posttraumatic Stress Disorder: A Nationwide Longitudinal Study." Psychosomatic Medicine 79(6): 664-669.	No Data on Migraine
Cheng, Y., et al. (2014). "Risk Factors of Post-Traumatic Stress Disorder (PTSD) after Wenchuan Earthquake: A Case Control Study." PloS One 9(5): e96644.	No Data on Migraine

Chibnall, J. T. and P. N. Duckro (1994). "Post-traumatic stress disorder in chronic post-traumatic headache patients." Headache 34(6): 357-361.	Sample size (<100 general; <50 PTSD; <50 non-PTSD)
Chung, M. C., et al. (2007). "Comorbidity and personality traits in patients with different levels of posttraumatic stress disorder following myocardial infarction." Psychiatry Research 152(2-3): 243-252.	No Data on Migraine
Clapp, J. D., et al. (2010). "Physical and psychosocial functioning following motor vehicle trauma: relationships with chronic pain, posttraumatic stress, and medication use." European Journal of Pain (London, England) 14(4): 418-425.	No Data on Migraine
Coppens, E., et al. (2017). "Prevalence and impact of childhood adversities and post-traumatic stress disorder in women with fibromyalgia and chronic widespread pain." European Journal of Pain (London, England) 21(9): 1582-1590.	No Data on Migraine
Coronas Borri, R., et al. (2001). "Factors associated to the development of Posttraumatic Stress Disorder." Actas Españolas de Psiquiatría: Acepsi 29(1): 10-12.	No Data on Migraine
Couch, J. and J. Hanas (2018). "Evaluation of post-traumatic (PT) chronic migraine (CM) in veterans of OEF/OIF campaigns using serum mass profiling to distinguish cm from other pt headaches by identification of peptide changes in serum." Neurology 90(15).	Review, Editorial, PhD Thesis, Conference Abstract
Couch, J. and K. Stewart (2017). "Migraine with Aura (MA) is the most common phenotype for Post-Traumatic Headache (PTH)." Neurology 88(16).	Review, Editorial, PhD Thesis, Conference Abstract
Couch, J. R. and K. E. Stewart (2016). "Headache Prevalence at 4-11 Years After Deployment-Related Traumatic Brain Injury in Veterans of Iraq and Afghanistan Wars and Comparison to Controls: A Matched Case-Controlled Study." Headache 56(6): 1004-1021.	No comparison of PTSD vs. non-PTSD
Couch, J. R. and K. Stewart (2017). "Headache severity in veterans deployed to afghanistan or iraq wars at 2-11 years after traumatic brain injury." Headache 57: 211-212.	Review, Editorial, PhD Thesis, Conference Abstract
Couch, J. R. and K. Stewart (2017). "Post-traumatic stress disorder and depression in relation to the different phenotypes of post-traumatic headache and comparison with matched controls." Cephalalgia 37(1): 154.	Review, Editorial, PhD Thesis, Conference Abstract
Couch, J. R., et al. (2018). "The relation of ptsd and severe depression to posttraumatic chronic migraine in veterans of Afghanistan and Iraq Wars." Headache 58: 198-199.	Review, Editorial, PhD Thesis, Conference Abstract
Cwikel, J., et al. (1997). "Two-year Follow-up Study of Stress-related Disorders among Immigrants to Israel from the Chernobyl Area." Environmental Health Perspectives 105(SUPPL. 6): 1545-1550.	Only Relative measures of Association (between PTSD and Migraine)
D'Amico, D., et al. (2018). "Multimorbidity in patients with chronic migraine and medication overuse headache." Acta Neurologica Scandinavica 138(6): 515-522.	No comparison of PTSD vs. non-PTSD
Danböck, S. K., et al. (2023). "Psychometric properties of the dissociative subtype of posttraumatic stress disorder scale: replication and extension in two German-speaking samples." Eur J Psychotraumatol 14(2): 2238492.	No Data on Migraine
Day, M. A., et al. (2022). "Transdiagnostic Cognitive Processes in Chronic Pain and Comorbid PTSD and Depression in Veterans." Annals of Behavioral Medicine 56(2): 157-167.	No Data on Migraine
De Leeuw, R., et al. (2005). "Prevalence of post-traumatic stress disorder symptoms in orofacial pain patients." Oral Surgery, Oral Medicine, Oral Pathology, Oral Radiology and Endodontics 99(5): 558-568.	No Data on Migraine

De Leeuw, R., et al. (2005). "Traumatic Stressors and Post-Traumatic Stress Disorder Symptoms in Headache Patients." Headache 45(10): 1365-1374.	Sample size (<100 general; <50 PTSD; <50 non-PTSD)
Dhangar, I., et al. (2023). "RWD118 Long-Term Neurologic Sequelae Among Patients with Varying COVID-19 Severity: An Administrative Claims Database Analysis." Value in Health 26(6): S383.	Review, Editorial, PhD Thesis, Conference Abstract
Dorflinger, L. M. and R. M. Masheb (2018). "PTSD is associated with emotional eating among veterans seeking treatment for overweight/obesity." Eat Behav 31: 8-11.	Sample size (<100 general; <50 PTSD; <50 non-PTSD)
Drost, L., et al. (2017). "Efficacy of different varieties of medical cannabis in relieving symptoms in ptsd patients." Supportive Care in Cancer 25(2): S223-S224.	No Data on Migraine
Dzubur Kulenovic, A., et al. (2009). "'The body keeps the score' revisited: Changes in lipid levels and coronary disease risk in army veterans suffering from chronic PTSD." European Psychiatry 24: S1242.	Review, Editorial, PhD Thesis, Conference Abstract
Ebrahimi, R., et al. (2021). "Association of Posttraumatic Stress Disorder and Incident Ischemic Heart Disease in Women Veterans." JAMA Cardiol 6(6): 642-651.	No Data on Migraine
Ebrahimi, R., et al. (2024). "Posttraumatic Stress Disorder Is Associated With Elevated Risk of Incident Stroke and Transient Ischemic Attack in Women Veterans." Journal of the American Heart Association 13(5): e033032.	No Data on Migraine
Eglinton, R. and M. C. Chung (2011). "The relationship between posttraumatic stress disorder, illness cognitions, defence styles, fatigue severity and psychological well-being in chronic fatigue syndrome." Psychiatry Research 188(2): 245-252.	No Data on Migraine
El-Gabalawy, R., et al. (2011). "Comorbid physical health conditions and anxiety disorders: a population-based exploration of prevalence and health outcomes among older adults." General Hospital Psychiatry 33(6): 556-564.	No comparison of PTSD vs. non-PTSD
Emrich, M., et al. (2024). "Associations of Posttraumatic Stress Disorder Symptom Clusters and Pain Interference in Post-9/11 Veterans: Exploring Sleep Impairment and Physical Activity as Underlying Mechanisms." International Journal of Behavioral Medicine.	No Data on Migraine
Engel, C. C., Jr., et al. (2000). "Relationship of Physical Symptoms to Posttraumatic Stress Disorder Among Veterans Seeking Care for Gulf War-Related Health Concerns." Psychosomatic Medicine 62(6): 739-745.	No Data on Migraine
Englert, R. M., et al. (2023). "Self-Reported Symptoms in U.S. Marines Following Blast- and Impact-Related Concussion." Military Medicine 188(7-8): e2118-e2125.	No Data on Migraine
Epstein, E. L., et al. (2019). "Posttraumatic stress disorder and traumatic brain Injury: Sex differences in veterans." Psychiatry Research 274: 105-111.	No Data on Migraine
Fellows, R. P., et al. (2015). "Psychological trauma exposure and co-morbid psychopathologies in HIV+Men and Women." Psychiatry Research 230(3): 770-776.	No Data on Migraine
Fenton, B. T., et al. (2020). "Prevalence of headache and comorbidities among men and women veterans across the veterans health administration-a 10-year cohort study." Headache 60: 58-59.	Review, Editorial, PhD Thesis, Conference Abstract
Fetzner, M. G., et al. (2012). "Similarities in specific Physical Health disorder prevalence among formerly deployed Canadian Forces Veterans with full and subsyndromal PTSD." Depression and Anxiety 29(11): 958-965.	No Data on Migraine

Finkel, A. G. (2022). "Headaches in Veterans: Different or the Same?" Neurology 99(18): 779-780.	Review, Editorial, PhD Thesis, Conference Abstract
Fischer, I. C., et al. (2023). "Employment status among US military veterans with a history of posttraumatic stress disorder: Results from the National Health and Resilience in Veterans Study." Journal of Traumatic Stress 36(6): 1167-1175.	No comparison of PTSD vs. no-PTSD
Fonda, J. R., et al. (2013). "Study of pseudobulbar Affect Symptoms in Veterans with mild Traumatic Brain Injury." Value in Health 16(3).	Review, Editorial, PhD Thesis, Conference Abstract
Fonda, J. R., et al. (2014). " Screening For PBA Symptoms using a Single Question versus a 7 Question Measure and Assessment of the Association of PBA Symptoms with HRQOL Burden." Value in Health 17(3).	Review, Editorial, PhD Thesis, Conference Abstract
Frayne, S. M., et al. (2004). "Burden of medical illness in women with depression and posttraumatic stress disorder." Archives of Internal Medicine 164(12): 1306-1312.	No Data on Migraine
Gaffey, A. E., et al. (2019). "Men and women veterans' military experiences and associated risk of insomnia." Sleep 42: A139-A140.	Review, Editorial, PhD Thesis, Conference Abstract
Gagnon-Sanschagrin, P., et al. (2022). "Identifying individuals with undiagnosed post-traumatic stress disorder in a large United States civilian population - a machine learning approach." BMC Psychiatry 22(1): 630.	No Data on Migraine
Gasperi, M., et al. (2021). "Pain and Trauma: The Role of Criterion A Trauma and Stressful Life Events in the Pain and PTSD Relationship." Journal of Pain 22(11): 1506-1517.	No comparison of PTSD vs. non-PTSD
Gasperi, M., et al. (2024). "Migraine Prevalence, Environmental Risk, and Comorbidities in Men and Women Veterans." Jama Network Open 7(3): E242299.	No comparison of PTSD vs. non-PTSD
Geisser, M. E., et al. (1996). "The relationship between symptoms of post-traumatic stress disorder and pain, affective disturbance and disability among patients with accident and non-accident related pain." Pain 66(2-3): 207-214.	No Data on Migraine
Gelaye, B., et al. (2013). "Migraine and psychiatric comorbidities among sub-saharan african adults." Headache 53(2): 310-321.	No comparison of PTSD vs. non-PTSD
Gelaye, B., et al. (2016). "Childhood Abuse, Intimate Partner Violence and Risk of Migraine Among Pregnant Women: An Epidemiologic Study." Headache 56(6): 976-986.	No comparison of PTSD vs. non-PTSD
Gibbons, S. K., et al. (2012). "Headaches in u.s. soldiers with concussion: A 12-month longitudinal study." Headache 52(5): 868.	Review, Editorial, PhD Thesis, Conference Abstract
Gill, G. K., et al. (2022). "Illness-induced post-traumatic stress disorder among Canadian Armed Forces Members and Veterans." Journal of Anxiety Disorders 86: 102472.	No comparison of PTSD vs. non-PTSD
Ginzburg, K. and Z. Solomon (2011). "Trajectories of stress reactions and somatization symptoms among war veterans: a 20-year longitudinal study." Psychological Medicine 41(2): 353-362.	No comparison of PTSD vs. non-PTSD
Glaesmer, H., et al. (2011). "The Association of Traumatic Experiences and Posttraumatic Stress Disorder With Physical Morbidity in Old Age: A German Population-Based Study." Psychosomatic Medicine 73(5): 401-406.	No Data on Migraine
Goldfinger, J. Z., et al. (2011). "Post-traumatic Stress Disorder in Stroke Survivors: Prevalence and Correlates." Journal of General Internal Medicine 26: S285.	Review, Editorial, PhD Thesis, Conference Abstract
Gradus, J. L., et al. (2022). "Pre-trauma predictors of severe psychiatric comorbidity 5 years following traumatic	No comparison of PTSD vs. non-PTSD

experiences." International Journal of Epidemiology 51(5): 1593-1603.	
Greggersen, W., et al. (2010). "Pain complaints in a sample of psychiatric inpatients." General Hospital Psychiatry 32(5): 509-513.	No comparison of PTSD vs. non-PTSD
Guàrdia, J., et al. (1998). "Desastres tecnológicos: estilo atribucional y estrés postraumático." Anuario de Psicología 29(3): 45-58.	No comparison of PTSD vs. non-PTSD
Guina, J., et al. (2018). "PTSD Symptom Severity, but Not Trauma Type, Predicts Mental Health Help-seeking in the Military." Journal of Psychiatric Practice 24(5): 310-316.	No comparison of PTSD vs. non-PTSD
Hall, K. S., et al. (2014). "PTSD is negatively associated with physical performance and physical function in older overweight military Veterans." Journal of Rehabilitation Research and Development 51(2): 285-295.	No Data on Migraine
Harner, H. M., et al. (2015). "Posttraumatic stress disorder in incarcerated women: A call for evidence-based treatment." Psychological Trauma: Theory, Research, Practice and Policy 7(1): 58-66.	No Data on Migraine
Henry, F. (2009). "La fibromyalgie : une forme atypique de stress post-traumatique ?" Douleurs : Evaluation - Diagnostic - Traitement 10(6): 326-327.	Review, Editorial, PhD Thesis, Conference Abstract
Hershey, L. A., et al. (2011). "Drugs that may cause headache or aggravate migraine in veterans referred to a transcranial doppler laboratory." Clinical Pharmacology and Therapeutics 89: S14.	Review, Editorial, PhD Thesis, Conference Abstract
Hilsenroth, M., Arsenault, L., & Sloan, P. (2005). Assessment of Combat-Related Stress and Physical Symptoms of Gulf War Veterans: Criterion Validity of Selected Hand Test Variables. Journal of Personality Assessment, 84(2), 155–162.	Sample size (<100 general; <50 PTSD; <50 non-PTSD)
Hinton, D. E., et al. (2018). "Migraine-Like Visual Auras Among Traumatized Cambodians with PTSD: Fear of Ghost Attack and Other Disasters." Culture, Medicine and Psychiatry 42(2): 244-277.	No comparison of PTSD vs. non-PTSD
Hruska, B., et al. (2023). "Examining the prevalence and health impairment associated with subthreshold PTSD symptoms (PTSS) among frontline healthcare workers during the COVID-19 pandemic." Journal of Psychiatric Research 158: 202-208.	No comparison of PTSD vs. non-PTSD
Husky, M. M., et al. (2018). "Gender differences in psychiatric and medical comorbidity with post-traumatic stress disorder." Comprehensive Psychiatry 84: 75-81.	No Data on Migraine
Iverson, K. M., et al. (2013). "Deployment-related traumatic brain injury among Operation Enduring Freedom/Operation Iraqi Freedom veterans: associations with mental and physical health by gender." J Womens Health (Larchmt) 22(3): 267-275.	No Data on Migraine
Jacob, L., et al. (2018). "Post-traumatic stress symptoms are associated with physical multimorbidity: Findings from the Adult Psychiatric Morbidity Survey 2007." Journal of Affective Disorders 232: 385-392.	No Data on Migraine
Jacobson, M. H., et al. (2018). "Longitudinal determinants of depression among World Trade Center Health Registry enrollees, 14-15 years after the 9/11 attacks." Journal of Affective Disorders 229: 483-490.	No Data on Migraine
Kimerling, R. (2004). "An investigation of sex differences in nonpsychiatric morbidity associated with posttraumatic stress disorder." J Am Med Womens Assoc (1972) 59(1): 43-47.	No Data on Migraine
Koo, B., et al. (2021). "Headache and pain multimorbidity in a national cohort of U.S. Veterans with cluster headache." Neurology 96(15 SUPPL 1).	Review, Editorial, PhD Thesis, Conference Abstract

Kronish, I. M., et al. (2012). "Post-traumatic stress disorder and medication adherence: results from the Mind Your Heart study." Journal of Psychiatric Research 46(12): 1595-1599.	No Data on Migraine
Lawrence-Wolff, K. M., et al. (2023). "Prevalence of Fibromyalgia Syndrome in Active-Duty Military Personnel." Arthritis Care & Research 75(3): 667-673.	No Data on Migraine
Leekoff, M., et al. (2022). "Impact of comorbid post traumatic stress disorder on multiple sclerosis in military veterans: A population-based cohort study." Multiple Sclerosis 28(8): 1257-1266.	No Data on Migraine
Li, H., et al. (2021). "Relationship Between Post-Traumatic Stress Disorder Symptoms and Chronic Pain-Related Symptom Domains Among Military Active Duty Service Members." Pain Medicine 22(12): 2876-2883.	No Data on Migraine
Li, M., et al. (2025). "Associations between post-traumatic stress disorder and neurological disorders: A genetic correlation and Mendelian randomization study." Journal of Affective Disorders 370: 547-556.	No comparison of PTSD vs. non-PTSD
Li, W. W., et al. (2024). "Bidirectional two-sample Mendelian randomization analysis identifies causal associations between migraine and five psychiatric disorders." Frontiers in Neurology 15: 1432966.	No comparison of PTSD vs. non-PTSD
Long, N., et al. (1992). "The health and mental health of New Zealand Vietnam war veterans with posttraumatic stress disorder." New Zealand Medical Journal 105(944): 417-419.	No Data on Migraine
Lopatkova, I. V., et al. (2018). "The interrelation of post-trauma stress disorders with reactive and personal anxiety." Electronic Journal of General Medicine 15(6).	No comparison of PTSD vs. no-PTSD
Lyons, M., et al. (2017). "The relationship of physical heath to mental health in the vietnam era twin study of aging (vetsa)." Twin Research and Human Genetics 20(6): 580.	Review, Editorial, PhD Thesis, Conference Abstract
Mainali, A., et al. (2024). "A comparative study of comorbidities, symptom profiles, and childhood trauma in PTSD and non-PTSD trauma patients at Oslo University Hospital." Nord J Psychiatry 78(8): 713-720.	No Data on Migraine
Manuel, J., et al. (2023). "Traumatic Events, Posttraumatic Stress Disorder, and Central Sensitization in Chronic Pain Patients of a German University Outpatient Pain Clinic." Psychosomatic Medicine 85(4): 351-357.	No Data on Migraine
Martalek, A., et al. (2024). "Distressing memories: A continuum from wellness to PTSD." Journal of Affective Disorders 363: 198-205.	No Data on Migraine
Mawanda, F., et al. (2017). "PTSD, Psychotropic Medication Use, and the Risk of Dementia Among US Veterans: A Retrospective Cohort Study." Journal of the American Geriatrics Society 65(5): 1043-1050.	No Data on Migraine
McDermott, M. J., et al. (2016). "The relation of PTSD symptoms to migraine and headache-related disability among substance dependent inpatients." Journal of Behavioral Medicine 39(2): 300-309.	No comparison of PTSD vs. non-PTSD
McFarlane, A. C., et al. (1994). "Physical symptoms in post-traumatic stress disorder." Journal of Psychosomatic Research 38(7): 715-726.	No Data on Migraine
McGinley, J. S., et al. (2023). "Longitudinal assessment of comorbidities and co-occurring conditions in adolescents with migraine: A secondary analysis of the National Longitudinal Study of Adolescent to Adult Health (Add Health)." Headache 63(2): 243-254.	No comparison of PTSD vs. non-PTSD

McLaren, G. (2012). "Health of women after wartime deployments: correlates of risk for selected medical conditions among females after initial and repeat deployments to Afghanistan and Iraq, active component, U.S. Armed Forces." MSMR 19(7): 2-10.	No comparison of PTSD vs. non-PTSD
McMillan, K. A. and G. J. G. Asmundson (2016). "PTSD, social anxiety disorder, and trauma: An examination of the influence of trauma type on comorbidity using a nationally representative sample." Psychiatry Research 246: 561-567.	No Data on Migraine
Mehta, D., et al. (2024). W2. Assessing the Comorbidity Between PTSD and Migraine Using Diverse Populations and Methods . 87: 101-102.	Review, Editorial, PhD Thesis, Conference Abstract
Merikangas, K. R., et al. (1990). "Migraine and psychopathology. Results of the Zurich cohort study of young adults." Archives of General Psychiatry 47(9): 849-853.	No comparison of PTSD vs. non-PTSD
Merlani, P., et al. (2009). "Post-traumatic stress disorder one year after ICU." Intensive Care Medicine 35: S106.	Review, Editorial, PhD Thesis, Conference Abstract
Milligan-Saville, J. S., et al. (2017). "The Amplification of Common Somatic Symptoms by Posttraumatic Stress Disorder in Firefighters." Journal of Traumatic Stress 30(2): 142-148.	No Data on Migraine
Miro, E., et al. (2020). "Clinical Manifestations of Trauma Exposure in Fibromyalgia: The Role of Anxiety in the Association Between Posttraumatic Stress Symptoms and Fibromyalgia Status." Journal of Traumatic Stress 33(6): 1082-1092.	No comparison of PTSD vs. non-PTSD
Moazen-Zadeh, E., et al. (2016). "Increased blood pressures in veterans with post traumatic stress disorder." International Journal of Psychiatry in Medicine 51(6): 576-586.	No Data on Migraine
Mostoufi, S. M. (2016). Posttraumatic stress symptoms and pain: Examining models of co-occurrence with twin analyses [ProQuest Information & Learning]. In Dissertation Abstracts International: Section B: The Sciences and Engineering (Vol. 76, Issue 11–B(E)).	Review, Editorial, PhD Thesis, Conference Abstract
Moye, J., et al. (2023). "Characteristics and Correlates of Ten-Year Trajectories of Posttraumatic Stress Symptoms in Older U.S. Military Veterans." American Journal of Geriatric Psychiatry 31(11): 889-901.	Sample size (<100 general; <50 PTSD; <50 non-PTSD)
Muhtz, C., et al. (2011). "[Long-term consequences of flight and expulsion in former refugee children]." Psychotherapie, Psychosomatik, Medizinische Psychologie 61(5): 233-238.	No Data on Migraine
Muhvić-Urek, M., et al. (2015). " Co-Occurrence of Chronic Head, Face and Neck Pain, and Depression in War Veterans With Post-Traumatic Stress Disorder." Acta Clinica Croatica 54(3): 266-271.	No Data on Migraine
Munyandamutsa, N., et al. (2012). "Mental and physical health in Rwanda 14 years after the genocide." Social Psychiatry and Psychiatric Epidemiology 47(11): 1753-1761.	No Data on Migraine
Ney, J. P., et al. (2020). "Survivors of military sexual trauma face increased risk of migraine headaches and worse headache care." Headache 60: 59.	Review, Editorial, PhD Thesis, Conference Abstract
Ney, J. P., et al. (2021). "Is post-traumatic headache in veterans different than traumatic brain injury with headache?" Headache 61(SUPPL 1): 51.	Review, Editorial, PhD Thesis, Conference Abstract
Niles, A. N., et al. (2015). "Anxiety and depressive symptoms and medical illness among adults with anxiety disorders." Journal of Psychosomatic Research 78(2): 109-115.	No comparison of PTSD vs. non-PTSD
Noteboom, A., et al. (2021). "The long-lasting impact of childhood trauma on adult chronic physical disorders." Journal of Psychiatric Research 136: 87-94.	No comparison of PTSD vs. non-PTSD

Ouimette, P., et al. (2006). "Health and well being of substance use disorder patients with and without posttraumatic stress disorder." Addictive Behaviors 31(8): 1415-1423.	No Data on Migraine
Patel, U. K., et al. (2020). "Triggers of Status Migrainosus and Higher Morbidity Amongst Migraineurs." Annals of Neurology 88(S25): S1-S280.	Review, Editorial, PhD Thesis, Conference Abstract
Patel, U., et al. (2020). "Evaluation of triggers of status migrainosus and its impact on morbidity." Neurology 94(15).	Review, Editorial, PhD Thesis, Conference Abstract
Peraica, T., et al. (2014). "Quality of life of Croatian veterans' wives and veterans with posttraumatic stress disorder." Health and Quality of Life Outcomes 12(1): 136.	No Data on Migraine
Perera, E., et al. (2021). "Chronic pain: The Canadian Armed Forces members and Veterans mental health follow-up survey." Journal of Military, Veteran and Family Health 7(S2): 29-42.	No comparison of PTSD vs. non-PTSD
Perez Benitez, C. I., et al. (2014). "Posttraumatic stress disorder in African Americans: a two year follow-up study." Psychiatry Research 220(1-2): 376-383.	No Data on Migraine
Peterlin, B. L., et al. (2009). "Posttraumatic stress disorder in migraine." Headache 49(4): 541-551.	No comparison of PTSD vs. non-PTSD
Peterlin, B. L., et al. (2012). "Men, women, and migraine: the role of sex, hormones, obesity, and PTSD." Journal of Family Practice 61(4 Suppl): S7-11.	Review, Editorial, PhD Thesis, Conference Abstract
Pietrzykowski, M. O., et al. (2024). "Co-Occurring Mental and Physical Health Conditions Among Older Adults With and Without Post-traumatic Stress Disorder: A Case Control Study." Journal of Geriatric Psychiatry and Neurology: 8919887241285558.	No Data on Migraine
Protuđer, M., et al. (2023). "Sexual Dysfunctions among Veterans with and without PTSD." Healthcare (Basel) 11(13).	No Data on Migraine
Ramon, A. E., et al. (2024). "Headache Disorders in VHA Primary Care: Prevalence, Psychiatric Comorbidity, and Health Care Utilization." Behavioral Medicine 50(4): 269-278.	No comparison of PTSD vs. non-PTSD
Reed, D. E., 2nd, et al. (2022). "Whole Health Use and Interest Across Veterans With Co-Occurring Chronic Pain and PTSD: An Examination of the 18 VA Medical Center Flagship Sites." Glob Adv Health Med 11: 21649561211065374.	No Data on Migraine
Rosendale, N., et al. (2022). "Migraine, Migraine Disability, Trauma, and Discrimination in Sexual and Gender Minority Individuals." Neurology 99(14): e1549-e1559.	No comparison of PTSD vs. non-PTSD
Rosenthal, J. F. and J. C. Erickson (2013). "Post-traumatic stress disorder in U.S. soldiers with post-traumatic headache." Headache 53(10): 1564-1572.	No Data on Migraine
Runnals, J. J., et al. (2013). "Self-Reported Pain Complaints among Afghanistan/Iraq Era Men and Women Veterans with Comorbid Posttraumatic Stress Disorder and Major Depressive Disorder." Pain Medicine 14(10): 1529-1533.	No Data on Migraine
Saunders, K., et al. (2008). "Impact of comorbidity on headache-related disability." Neurology 70(7): 538-547.	Only Relative Measures
Schlenger, W. E., et al. (2015). "A Prospective Study of Mortality and Trauma-Related Risk Factors Among a Nationally Representative Sample of Vietnam Veterans." American Journal of Epidemiology 182(12): 980-990.	No Data on Migraine
Scott, G. D., et al. (2023). "Lifelong Association of Disorders Related to Military Trauma with Subsequent Parkinson's Disease." Movement Disorders 38(8): 1483-1492.	Only Relative measures of Association (between PTSD and Migraine)

Senaratne, R., et al. (2010). "The Prevalence of Migraine Headaches in an Anxiety Disorders Clinic Sample." CNS Neuroscience & Therapeutics 16(2): 76-82.	No comparison of PTSD vs. non-PTSD
Serier, K. N., et al. (2022). "Analysis of the bidirectional relationships between posttraumatic stress and depression symptoms with physical health functioning in post-9/11 veteran men and women deployed to a war zone." Journal of Psychosomatic Research 162: 111034.	No comparison of PTSD vs. non-PTSD
Shipherd, J. C., et al. (2007). "Veterans seeking treatment for posttraumatic stress disorder: what about comorbid chronic pain?" Journal of Rehabilitation Research and Development 44(2): 153-166.	No comparison of PTSD vs. non-PTSD
Shor, R., et al. (2023). "The transition to civilian life: Impact of comorbid PTSD, chronic pain, and sleep disturbance on veterans' social functioning and suicidal ideation." Psychological Trauma: Theory, Research, Practice and Policy 15(8): 1315-1323.	No Data on Migraine
Sico, J. J., et al. (2022). "Characteristics and Gender Differences of Headache in the Veterans Health Administration: A National Cohort Study, Fiscal Year 2008-2019." Neurology 99(18): e1993-e2005.	No comparison of PTSD vs. non-PTSD
Sloan, P., et al. (2005). "Impact of Event Scale Prediction of DSM-IV PTSD and Physical Symptoms in Gulf War Veterans." Stress, Trauma, and Crisis 8(4): 215-228.	Sample size (<100 general; <50 PTSD; <50 non-PTSD)
Solomon, Z. (1988). "Somatic Complaints, Stress Reaction, and Posttraumatic Stress Disorder: A Three-Year Follow-up Study." Behavioral Medicine 14(4): 179-185.	No comparison of PTSD vs. non-PTSD
Solomon, Z., et al. (1987). "A two year follow-up of somatic complaints among Israeli combat stress reaction casualties." Journal of Psychosomatic Research 31(4): 463-469.	No comparison of PTSD vs. non-PTSD
Solomon, Z., et al. (2009). "Subjective age, PTSD and physical health among war veterans." Aging & Mental Health 13(3): 405-413.	No Data on Migraine
Solomon, Z., et al. (2014). "The long-term implications of war captivity for mortality and health." Journal of Behavioral Medicine 37(5): 849-859.	No comparison of PTSD vs. non-PTSD
Sommer, J. L., et al. (2019). "Understanding the association between posttraumatic stress disorder characteristics and physical health conditions: A population-based study." Journal of Psychosomatic Research 126: 109776.	No Data on Migraine
Sommer, J. L., et al. (2021). "Associations between physical health conditions and posttraumatic stress disorder according to age." Aging & Mental Health 25(2): 234-242.	No Data on Migraine
Sommer, J. L., et al. (2022). "Associations between courses of posttraumatic stress disorder and physical health conditions among Canadian military personnel." Journal of Anxiety Disorders 87: 102543.	No Data on Migraine
Spitzer, C., et al. (2010). "Association of posttraumatic stress disorder with low-grade elevation of C-reactive protein: Evidence from the general population." Journal of Psychiatric Research 44(1): 15-21.	No Data on Migraine
Theeler, B. J. and J. C. Erickson (2009). "Mild head trauma and chronic headaches in returning US soldiers." Headache 49(4): 529-534.	No comparison of PTSD vs. non-PTSD
Thomas, M. M., et al. (2017). "Mental and Physical Health Conditions in US Combat Veterans: Results From the National Health and Resilience in Veterans Study." Primary Care Companion to CNS Disorders 19(3).	No comparison of PTSD vs. non-PTSD

Trafton, J. A., et al. (2006). "Opioid substitution treatment reduces substance use equivalently in patients with and without posttraumatic stress disorder." Journal of Studies on Alcohol 67(2): 228-235.	No Data on Migraine
Uhac, I., et al. (2006). "The Prevalence of Temporomandibular Disorders in War Veterans with Post-Traumatic Stress Disorder." Military Medicine 171(11): 1147-1149.	No Data on Migraine
Van Der Merwe, J., et al. (2021). "Military veterans with and without post-traumatic stress disorder: results from a chronic pain management programme." Scand J Pain 21(3): 560-568.	No Data on Migraine
Vandiver, R. A., et al. (2023). "PTSD symptom severity mediates the impact of war zone stress exposure on postdeployment physical health: The Fort Devens Gulf War veterans cohort." Psychological Trauma: Theory, Research, Practice and Policy 15(4): 681-689.	No comparison of PTSD vs. non-PTSD
Vij, B., et al. (2015). "(210) Prevalence of migraine headaches in patients with Fibromyalgia." The Journal of Pain 16(4).	Review, Editorial, PhD Thesis, Conference Abstract
Vij, B., et al. (2015). "Frequency of Migraine Headaches in Patients With Fibromyalgia." Headache 55(6): 860-865.	No comparison of PTSD vs. non-PTSD
Walsh, E., et al. (2017). "The Importance of Assessing for Childhood Abuse and Lifetime PTSD in Bariatric Surgery Candidates." Journal of Clinical Psychology in Medical Settings 24(3-4): 341-354.	No Data on Migraine
Wang, T. Y., et al. (2016). "Risk for developing dementia among patients with posttraumatic stress disorder: A nationwide longitudinal study." Journal of Affective Disorders 205: 306-310.	No Data on Migraine
Weisberg, R. B., et al. (2002). "Nonpsychiatric illness among primary care patients with trauma histories and posttraumatic stress disorder." Psychiatric Services 53(7): 848-854.	No Data on Migraine
Wiley, F. (2020). "Pharmacists can help manage migraines in patients with psychiatric conditions add value by optimizing medications, decreasing pill burden." Drug Topics Journal: Vol.165, Issue 11	Review, Editorial, PhD Thesis, Conference Abstract
Yimer, G. M., et al. (2023). "Determinants of post-traumatic stress disorder among survivors of road traffic accidents in dessie comprehensive specialized hospital North-East Ethiopia." BMC Psychiatry 23(1): 218.	No Data on Migraine
Yu, S., et al. (2016). "Impact of 9/11-related chronic conditions and PTSD comorbidity on early retirement and job loss among World Trade Center disaster rescue and recovery workers." American Journal of Industrial Medicine 59(9): 731-741.	No Data on Migraine
Yue, J. K., et al. (2018). "Preinjury employment status as a risk factor for symptomatology and disability in mild traumatic brain injury: A TRACK-TBI analysis." Neuro Rehabilitation 43(2): 169-182.	No comparison of PTSD vs. non-PTSD
Zatzick, D. F., et al. (1997). "Posttraumatic stress disorder and functioning and quality of life outcomes in a nationally representative sample of male Vietnam veterans." American Journal of Psychiatry 154(12): 1690-1695.	No Data on Migraine
Zatzick, D. F., et al. (2010). "Multisite investigation of traumatic brain injuries, posttraumatic stress disorder, and self-reported health and cognitive impairments." Archives of General Psychiatry 67(12): 1291-1300.	No Data on Migraine
Zhang, W., et al. (2004). "Posttraumatic stress disorder in callers to the Anxiety Disorders Association of America." Depression and Anxiety 19(2): 96-104.	No Data on Migraine
Zhang, Y., et al. (2019). "Post-traumatic stress disorder in living donors after pediatric liver transplantation: A cross-sectional investigation study." Medicine (Baltimore) 98(20): e15565.	No Data on Migraine

Zhou, H., et al. (2016). "The Effect of Maternal Death on the Health of the Husband and Children in a Rural Area of China: A Prospective Cohort Study." PloS One 11(6): e0157122.	No comparison of PTSD vs. non-PTSD
Ziobrowski, H., et al. (2017). "Gender differences in mental and physical health conditions in U.S. veterans: Results from the National Health and Resilience in Veterans Study." Journal of Psychosomatic Research 101: 110-113.	No comparison of PTSD vs. non-PTSD

The assignment of the exclusion was done hierarchically:

1. Review, Editorial, PhD Thesis, Conference Abstract
2. No comparison of PTSD vs. non-PTSD
3. Sample size (<100 general; <50 PTSD; < 50 non-PTSD)
4. No data on migraine
5. Only relative measures of association

References

1. Vun E, Turner S, Sareen J, Mota N, Afifi TO, El-Gabalawy R. Prevalence of comorbid chronic pain and mental health conditions in Canadian Armed Forces active personnel: analysis of a cross-sectional survey. *CMAJ Open*. 2018;6(4):E528-E536. doi:10.9778/cmajo.20180093
2. Smitherman TA, Kolivas ED. Trauma Exposure versus Posttraumatic Stress Disorder: Relative Associations With Migraine. *Headache*. 2013;53(5):775-786. doi:10.1111/head.12063
3. Rao AS, Scher AI, Vieira RV, Merikangas KR, Metti AL, Peterlin BL. The Impact of Post-Traumatic Stress Disorder on the Burden of Migraine: Results From the National Comorbidity Survey-Replication. *Headache*. 2015;55(10):1323-1341. doi:10.1111/head.12698
4. Herbert MS, Merritt VC, Afari N, Gasperi M. Cognitive symptoms in veterans with migraine or traumatic brain injury: A Million Veteran Program study. *Headache*. 2025;65(3):430-438. doi:10.1111/head.14815. Epub 2024 Aug 28.
5. Gasperi M, Panizzon M, Goldberg J, Buchwald D, Afari N. Posttraumatic Stress Disorder and Chronic Pain Conditions in Men: A Twin Study. *Psychosom Med*. 2021;83(2):109-117. doi:10.1097/PSY.0000000000000899
6. Friedman LE, Aponte C, Hernandez RP, et al. Migraine and the risk of post-traumatic stress disorder among a cohort of pregnant women. *J Headache Pain*. 2017;18(67. doi:10.1186/s10194-017-0775-5
7. Friedman LE, Zhong Q-Y, Gelaye B, Williams MA, Peterlin BL. Association Between Migraine and Suicidal Behaviors: A Nationwide Study in the USA. *Headache*. 2018;58(3):371-380. doi:10.1111/head.13235
8. El-Gabalawy R, Blaney C, Tsai J, Sumner JA, Pietrzak RH. Physical health conditions associated with full and subthreshold PTSD in U.S. military veterans: Results from the National Health and Resilience in Veterans Study. *J Affect Disord*. 2018;227(849-853. doi:10.1016/j.jad.2017.11.058
9. Barer Y, Chodick G, Chodick NG, Gurevich T. Risk of Parkinson Disease Among Adults With vs Without Posttraumatic Stress Disorder. *JAMA Netw Open*. 2022;5(8):e2225445. doi:10.1001/jamanetworkopen.2022.25445
10. Crowe HM, Sampson L, Purdue-Smithe AC, Rexrode KM, Koenen KC, Rich-Edwards JW. Bidirectional analysis of the association between migraine and post-traumatic stress disorder in Nurses' Health Study II. *Epidemiol Psychiatr Sci*. 2024;33:e76 doi:10.1017/s2045796024000799
11. Huang M-H, Chan Y-LE, Hsu J-W, et al. Risk of Developing Migraine Among Patients with Posttraumatic Stress Disorder: A Nationwide Longitudinal Study. *Taiwan J Psychiatry*. 2019;33(4):192-197. doi:10.4103/tpsy.Tpsy_40_19
